# Clemastine alleviates Parkinson’s disease pathology by activating the AMPK/mTOR pathway and enhancing autophagic flux

**DOI:** 10.3389/fnagi.2026.1915475

**Published:** 2026-09-29

**Authors:** Ting Fan, Caihui Wei, Wenzhi Chen, Shu Li, Junling Wang, Yi Lu, Renshi Xu

**Affiliations:** 1 School of Clinical Medicine, Jiangxi University of Traditional Chinese Medicine, Nanchang, Jiangxi, China; 2 Department of Neurology, Jiangxi Provincial People’s Hospital, The First Affiliated Hospital of Nanchang Medical College, Nanchang, Jiangxi, China; 3 Department of Neurology, National Regional Center for Neurological Diseases, Xiangya Hospital of Central South University Jiangxi Hospital, Nanchang, Jiangxi, China; 4 Department of Cadres Health Care, The First Affiliated Hospital, Jiangxi Medical College, Nanchang University, Nanchang, Jiangxi, China

**Keywords:** AMPK/mTOR pathway, autophagy, bioinformatics, clemastine, drug repurposing, neuroprotection, Parkinson’s disease, α-Synuclein

## Abstract

Parkinson’s disease (PD) is a debilitating neurodegenerative disorder with no available cure, underscoring the urgent need for novel therapeutics. This study employed the comprehensive bioinformatic analyses and the experimental models to investigate the potential of clemastine (CLE) for PD treatment. Bioinformatics identified 12 distinct cell subtypes and 577 differentially expressed genes in dopaminergic neurons (DaNs), along with 167 potential PD-relevant CLE targets. A significant correlation was found between Braak stage and autophagy-related gene expression in the substantia nigra. In MPP^+^-induced PC12 cell model, CLE treatment increased AMPK phosphorylation and decreased mTOR phosphorylation at Ser2448, suggesting the activation of AMPK and reduced mTOR activity, which was accompanied by the reduced levels of α-synuclein (α-syn) and p62, increased lysosomal (LAMP1) and autophagosomal (LC3) markers, and enhanced cell viability. CLE increased the LC3-II/LC3-I ratio even in the presence of bafilomycin A1, indicating enhanced autophagosome synthesis. Conversely, the AMPK inhibitor dorsomorphin blocked CLE effects, restoring mTOR phosphorylation and α-syn levels. In the chronic MPTP-induced mouse model, CLE administration increased the phosphorylation of AMPK, increased the LC3-II/LC3-I ratio, and decreased mTOR phosphorylation, p62, and α-syn levels. Immunofluorescence revealed the increased DaNs (tyrosine hydroxylase-positive) and LC3 puncta, alongside reduced the phosphorylated α-syn (p-S129). These cellular and molecular improvements correlated with the alleviation of both motor (rotarod test) and non-motor (fecal pellet output) deficits. Collectively, our findings suggest that CLE alleviates PD pathology by increasing AMPK phosphorylation and decreasing mTOR phosphorylation at Ser2448, suggesting the activation of AMPK pathway and reduced mTOR activity, thereby enhancing the autophagic flux by promoting both the synthesis and degradation of autophagosomes. Subsequently it facilitates the clearance of pathological α-syn, improves the neuronal survival, and ameliorates the behavioral symptoms. We propose CLE as a promising therapeutic candidate for PD via autophagy modulation.

## Introduction

Parkinson’s disease (PD) is a common neurodegenerative disorder that primarily affects middle-aged and elderly individuals. The disease is characterized by the progressive degeneration of dopaminergic neurons (DaNs) in the substantia nigra and the formation of Lewy bodies. PD is primarily considered a movement disorder, with three cardinal symptoms: tremor, rigidity, and bradykinesia. A recent revision of diagnostic criteria has excluded postural instability as a fourth hallmark (Tysnes and Storstein, 2017). Additionally, non-motor symptoms such as depression, constipation, pain, genitourinary issues, sleep disorders, as well as cognitive and psychiatric impairments, have garnered increasing attention (Pfeiffer, 2016; Goldman, 2025). PD significantly impairs the quality of life and reduces life expectancy in the elderly, placing substantial emotional and economic burdens on families and society.

According to current epidemiological studies, the number of PD cases is projected to rise to between 8.7 and 9.3 million by 2030 in the top five Western European countries and the top ten most populated nations worldwide (Dorsey et al., 2007). This increase in PD incidence correlates with the aging global population. Age is the primary risk factor for PD, with prevalence rising significantly after the age of 60. Only 5% to 10% of cases are diagnosed before the age of 50. Across all age groups, men are estimated to be 1.5 times more likely to develop PD than women (Tysnes and Storstein, 2017; Ben-Shlomo et al., 2024).

Although the exact pathogenesis of PD remains incompletely understood, several mechanisms have been implicated, including oxidative stress (Chen et al., 2025), mitochondrial dysfunction (Moradi Vastegani et al., 2023), DNA fragmentation (Gogna et al., 2024), protein misfolding (Utami et al., 2025), endoplasmic reticulum stress (Mou et al., 2020), microglial activation (Ho, 2019), and apoptosis (Lev et al., 2003). Lewy bodies are the neuronal inclusions composed predominantly of misfolded and aggregated alpha-synuclein (α-syn) (Mehra et al., 2019). α-Syn is a pathological hallmark of both sporadic and familial PD, as well as other synucleinopathies. It is highly expressed in the brain, mainly in neurons, but also present in other tissues such as red blood cells (Kluge et al., 2022; Li et al., 2022).

Multiple mechanisms underlie the α-syn-mediated neurodegeneration, including the disrupted membrane permeability (Surguchev and Surguchov, 2015), the dysfunction of the endoplasmic reticulum (Gallegos et al., 2015) and Golgi apparatus (Stefanis, 2012; Wong and Krainc, 2017; Paiva et al., 2018), impaired protein degradation pathways (Wang et al., 2024a), mitochondrial damage (Mingo et al., 2025), endolysosomal system dysfunction (Vidyadhara et al., 2019; Teixeira et al., 2021), and neuroinflammation (Rocha et al., 2018). A central hydrophobic region (residues 71–82) of α-syn is particularly prone to misfolding and aggregation (Jha et al., 2018). α-Syn is degraded primarily via the ubiquitin-proteasome system and the autophagy-lysosome pathway (Xilouri et al., 2013). The former degrades short-lived soluble proteins, while the latter clears long-lived macromolecules, cellular components, and damaged organelles (Kocaturk and Gozuacik, 2018; Byrnes et al., 2022; Fleming et al., 2022). Dysfunction in these systems leads to α-syn accumulation, disrupting cellular homeostasis and contributing to PD pathogenesis (Xilouri et al., 2013).

Autophagy is typically upregulated under cellular stress (Peker and Gozuacik, 2020). Substrates containing KFERQ motifs are recognized by chaperone complexes and delivered to the lysosomal receptor LAMP-2A, triggering chaperone-mediated autophagy (Yao and Shen, 2023). This pathway plays a significant role in α-syn metabolism, though other autophagic mechanisms may also be involved (Arias-Carrión et al., 2025).

Current PD treatments – including medication, surgery, and supportive therapies – only alleviate symptoms rather than halting disease progression. Levodopa-based therapies, dopamine agonists, MAO-B inhibitors, and anticholinergics are commonly used to restore dopaminergic function. However, their efficacy often declines over time, and side effects are common. Deep brain stimulation is available for select patients but is not universally applicable (Veilleux Carpentier and Okun, 2025). The adjunctive approaches such as exercise, rehabilitation, and the psychological support provide the limited symptomatic relief (Armstrong and Okun, 2020). Thus, there is an urgent need for novel therapeutic strategies.

Recent advances in high-throughput screening and molecular docking have accelerated drug discovery and repurposing efforts (Zhao et al., 2025). Molecular docking simulates interactions between ligands and target proteins, aiding in the identification and optimization of drug candidates – particularly in neurodegenerative, infectious, and oncological research (Ke et al., 2016; Wang et al., 2024b; Dutta et al., 2025; Liu et al., 2025). This computational approach predicts binding affinity and the mode of action without prior knowledge of ligand chemistry (Pinzi and Rastelli, 2019; Stanzione et al., 2021; Zong et al., 2025). However, its accuracy depends on the availability of high-quality protein structures. The Protein Data Bank (PDB) is a major resource but contains incomplete entries and limited structural coverage (Burley et al., 2022), complicating large-scale docking studies.

The AlphaFold database has emerged as a transformative tool, offering over 200 million high-accuracy protein structure predictions (Jumper et al., 2021; Varadi et al., 2022). It enables large-scale structural bioinformatics and facilitates drug target screening and mechanistic studies (Ruff and Pappu, 2021). RNA sequencing also plays a crucial role in drug development by assessing transcriptomic responses to compounds, aiding in toxicity evaluation, dose optimization, target identification, and resistance mechanism analysis (Van de Sande et al., 2023; Wang et al., 2023). Integrating transcriptomics with molecular docking allows for the systematic evaluation of drug–target interactions (Wu et al., 2024). Successful examples include HIV protease inhibitors (Turkovic et al., 2020), antidiabetic agents like sitagliptin, and anticancer drugs such as rosutinib (Pinzi and Rastelli, 2019).

Clemastine (CLE), an H1-antihistamine used for allergic rhinitis, has shown promise in remyelination and neuroprotection. It promotes oligodendrocyte differentiation and myelination (Xie et al., 2021), improves memory in Alzheimer’s disease models (Chen et al., 2021), and facilitates myelin repair in multiple sclerosis and autism models (Palma et al., 2022; Bohlen et al., 2023). Its mechanism may involve muscarinic M1 receptor antagonism (Chen et al., 2021). CLE also exhibits anti-inflammatory properties. It modulates cytokines such as IL-1β and TNF-α (Su et al., 2018), and may inhibit TLR4/NF-κB signaling (Yuan et al., 2020). Additionally, CLE suppresses mast cell degranulation, reducing histamine and protease release that exacerbate neuroinflammation (Wu et al., 2021). It also enhances the autophagic clearance of protein aggregates in PD cellular models (Kataura et al., 2021). Based on these findings, this study aims to further investigate the therapeutic potential of CLE in PD and elucidate its underlying mechanisms, offering a promising candidate for drug repurposing in PD (Figure 1).

**FIGURE 1:**
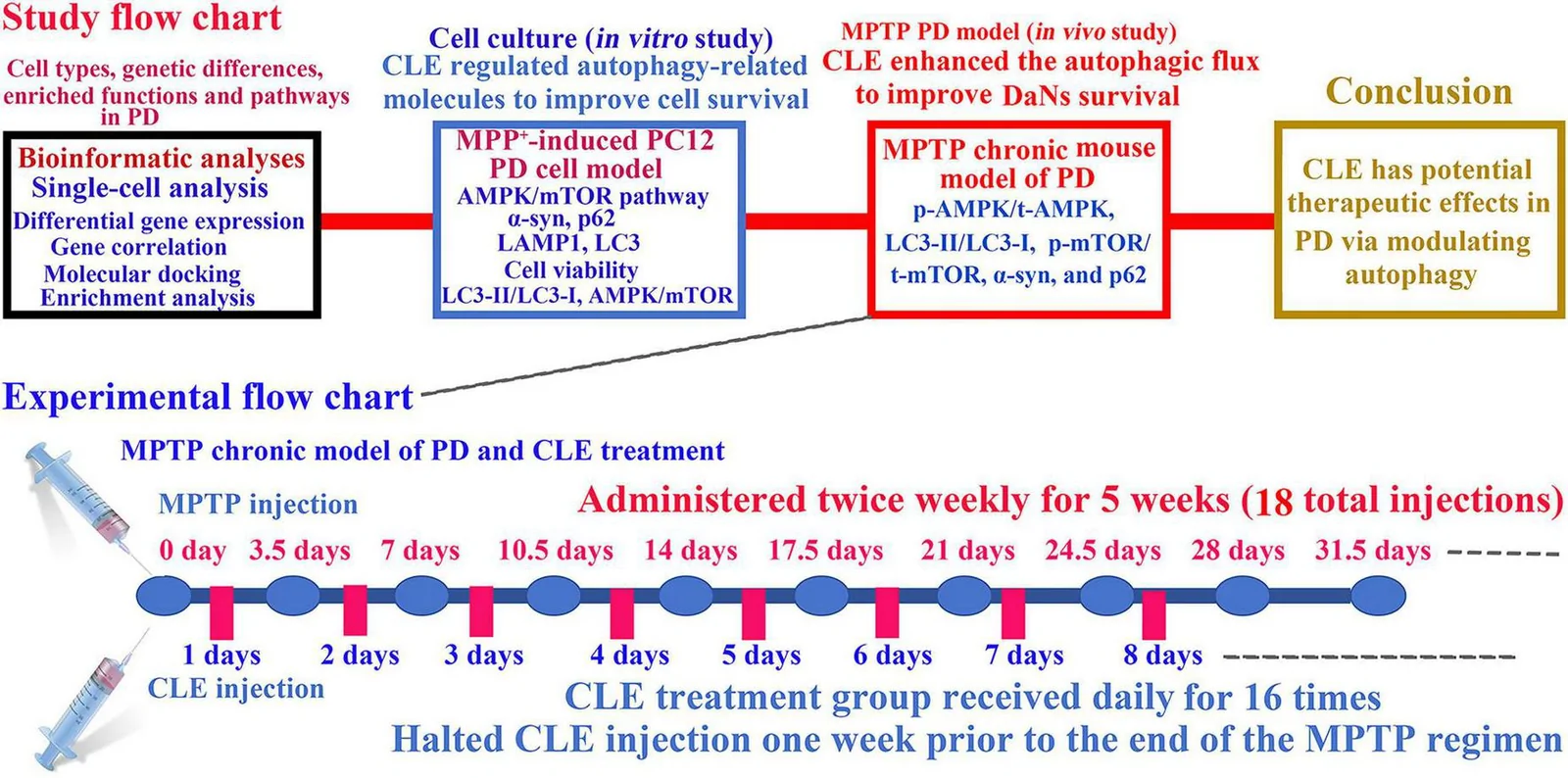
Study and experimental flow chart. This Figure illustrates the overall experimental design and workflow of the study. The study integrates bioinformatic analyses with *in vitro* and *in vivo* experimental validations. Bioinformatic approaches included the single-cell transcriptome analysis of Parkinson’s disease (PD) patient midbrains to identify differentially expressed genes (DEGs) in dopaminergic neurons (DaNs) and cell type alterations, molecular docking between clemastine (CLE) and 22,618 proteins to identify potential targets, and correlation analysis between Braak staging and autophagy gene expression. The intersection of CLE targets and PD-related DEGs was used to predict the CLE’s mechanism of action, focusing on the AMPK/mTOR pathway and autophagy. These predictions were tested experimentally using an MPP^+^-induced PC12 cell model and a chronic MPTP-induced mouse model of PD. Endpoints assessed included protein expression (western blot and immunofluorescence), autophagic flux, α-synuclein (α-syn) and phosphorylated α-syn (p-α-syn) levels, dopaminergic neuron survival, motor function (rotarod test), and non-motor function (fecal pellet analysis). The integrative approach aims to demonstrate that CLE alleviates PD pathology by activating the AMPK/mTOR pathway, enhancing autophagic flux, and promoting the clearance of pathological α-syn. PD, Parkinson’s disease; CLE, Clemastine; MPTP, 1-Methyl-4-phenyl-1,2,3,6-tetrahydropyridine; MPP^+^, 1-methyl-4-phenylpyridinium; DEGs, differentially expressed genes; DaNs, dopaminergic neurons; GO, Gene Ontology; KEGG, Kyoto Encyclopedia of Genes and Genomes; AMPK, AMP-activated protein kinase; mTOR, mammalian target of rapamycin; α-syn, α-synuclein; p-α-syn: phosphorylated α-synuclein.

## Methods

### Gene expression profile analysis of PD

To obtain gene expression profile data for subsequent differential gene analysis, single-cell gene expression data from PD patients were downloaded from the Gene Expression Omnibus (GEO) database in the NCBI database, using the accession number GSE157783. Due to the high cost of single-cell sequencing and the scarcity of human midbrain specimens, this dataset includes single-cell transcriptome data from the midbrains of only 5 PD cases and 6 age-matched healthy controls (HCs) from the UK PD Brain Bank and Newcastle Brain Resource Center. The five PD patients in the case group met the diagnostic criteria of the UK PD Brain Bank, providing an average of 3,800 cells per sample and having an average age of 77 years (the standard error of mean, SEM = 3). The HCs group provided an average of 3,738 cells per sample, with an average age of 81 years (SEM = 4). The post-mortem sampling time interval was similar between the PD and HC groups (approximately 22 h for PD and 16 h for HCs).

### Processing of single-cell transcriptome data

The PD single-cell transcriptome data were processed using the Seurat software package in R. Single-cell RNA sequencing data analysis was performed by generating barcode.tsv, genes.tsv, and matrix.mtx files from the source files, importing the data, and creating Seurat objects. The Seurat package was further used for quality control and data processing. Based on the number of unique genes, the total number of molecules, and the proportion of mitochondrial genes detected per cell, low-quality cells such as empty droplets, doublets, multiplets, or dead cells were filtered out. Gene expression for each cell was standardized and normalized to ensure the comparability of expression levels across cells. To highlight biological signals for Uniform Manifold Approximation and Projection (UMAP) analysis, downstream processes including the identification of highly variable genes, the determination of dimensionality reduction parameters for UMAP, and the visualization of high-dimensional data in two dimensions were performed. The Louvain algorithm was applied for the unsupervised clustering of cells based on UMAP results to identify distinct cell types or subtypes. Differential gene expression analysis was conducted between each cluster and all remaining cells, and the results were compared with known cell marker genes to annotate cell types. Differential gene expression analysis was performed using the FindMarkers function in Seurat, which employs a Wilcoxon rank-sum test comparing expression between PD and HC groups. We acknowledge that this approach treats individual cells as the statistical unit; a donor-level/pseudobulk approach would provide more robust statistical inference and should be considered in future studies.

### KEGG pathway enrichment analysis

The Kyoto Encyclopedia of Genes and Genomes (KEGG) was used to investigate advanced biological system functions, such as metabolic processes and signaling pathways. The clusterProfiler package (version 4.0.5) in R was used for KEGG pathway enrichment analysis, providing a universal interface for functional annotation of genes from various sources. Terms with an adjusted p-value (p.adjust) < 0.05 were considered significantly enriched. Detailed procedures followed our previous study (Wei et al., 2022a).

### GO functional enrichment analysis

Gene Ontology (GO) analysis was applied in areas such as gene expression profiling, protein interaction network analysis, and novel gene function prediction. Genes and gene products were annotated using GO terms to explore their biological significance. The clusterProfiler package in R was used for GO enrichment analysis, with terms having a q-value < 0.05 considered significantly enriched.

### Correlation analysis between Braak staging and autophagy gene expression in the substantia nigra of PD patients

The relationship between Braak staging and the expression of autophagy genes in the substantia nigra was analyzed using R and the rcorr function from the Hmisc package. A total of 208 common autophagy genes were obtained from the Human Autophagy Database. The GSE49036 dataset was downloaded from the GEO database, containing transcriptome data from the substantia nigra of 20 PD patients and 8 age-matched HCs. Among these, the Braak stage was 0 for all 8 HCs, 1–2 for 5 PD patients, 3–4 for 7 PD patients, and 5–6 for 8 PD patients. Gene ID annotation, data standardization, and gene expression matrix extraction were performed. The preprocessed, standardized, and normalized expression matrix data were subjected to Spearman correlation analysis to evaluate nonlinear correlations between indicators, with correlation coefficients ranging from −1 to 1.

### Molecular docking analysis

Ligand-protein docking analysis was performed to predict the binding mode between ligands and known three-dimensional protein structures, serving as a key method in computer-aided drug design. This technique allows for the virtual screening of large compound libraries, the ranking of results, and the generation of structural hypotheses on ligand-target inhibition. Autodock Vina (version 1.2.0) is a semi-flexible docking program that allows ligand flexibility while keeping the protein rigid. With CLE having 6 rotatable bonds, docking accuracy was high and computational speed was fast. Autodock Vina (version 1.2.0) was used for molecular docking, following procedures described in our previous study (Wei et al., 2022b).

### Acquisition and preparation of protein and compound data

Protein structures were obtained from the AlphaFold database stored at EMBL-EBI. Data for humans (version 3) were downloaded, comprising 20,594 compressed PDB format files. The SDF file for the chemical spatial structure of CLE was downloaded from PubChem.

### Preparation of receptors

Proteins used in molecular docking were designated as receptors. Since protein crystals may contain ligands, cofactors, ions, and water molecules, each protein structure was preprocessed to remove these impurities. On Python 3.8, the prepare_receptor.py program in AutoDockTools (MGLTools version 1.5.7) was used to add hydrogen atoms, generating PDBQT files containing only polar hydrogens and partial charges. Docking box parameters for each protein were calculated using the PandasPdb function in the biopandas package (version 0.4.1).

### Preparation of ligands

Small molecules in docking were set as ligands. As the CLE SDF file might not include hydrogen atoms, a pH value was defined prior to hydrogenation. On Python 3.8, the prepare_ligand.pyprogram in AutoDockTools was used to add hydrogens to the CLE SDF file and output it in PDBQT format for Autodock Vina.

### Protein-ligand docking

Autodock Vina was used on Python 3.8 for molecular docking. The storage locations for the CLE and protein PDBQT files were specified, and the box parameters for each protein were set. Exhaustiveness was set to 32. Docking results were evaluated based on binding energy, with lower (more negative) values indicating more stable binding. Binding energy < 0 kcal/mol indicates spontaneous binding; < −5 kcal/mol indicates good binding activity; and < −7 kcal/mol indicates strong binding activity. GO and KEGG enrichment analyses were performed on proteins found to interact with CLE.

### Potential targets of CLE in PD

The gene names of proteins interacting with CLE and differentially expressed genes (DEGs) in dopamine neurons (DaNs) in PD were standardized using the ID mapping function on the UniProt website. The intersection of these two gene lists was identified as the potential targets of CLE in PD. GO and KEGG enrichment analyses were performed to clarify the biological functions of CLE in PD.

### Cell culture and treatment

PC12 cells (Cat No. TCR9, RRID:CVCL_F659; purchased from the cell bank of the Chinese Academy of Sciences) were cultured in high-glucose Dulbecco’s Modified Eagle Medium (DMEM; Gibco) supplemented with 10% fetal bovine serum (FBS; Gibco) and 1% penicillin-streptomycin. Cells were divided into a control group (CTRL (Selleck)) and a PD model group (1-methyl-4-phenylpyridinium (MPP^++^; Selleck)). The CTRL group was treated with normal saline (NS; volume equal to that of MPP^+^), while the PD model group was treated with MPP^+^ at a final concentration of 1 mM for 24 h. Cells in both CTRL and MPP^+^ groups were subsequently treated with CLE, Baf-A1 (Selleck), or dorsomorphin (DOR; Selleck) according to the experimental design. Specifically, CLE was administered at a final concentration of 10 μM for 24 h, concurrent with MPP^+^ treatment. The CLE dose of 10 μM for *in vitro* studies was selected based on previous studies demonstrating effective autophagy induction and neuroprotection in PC12 cells (Kataura et al., 2021). Bafilomycin A1 was administered at 100 nM for 4 h prior to cell collection. Dorsomorphin was administered at 5 μM for 2 h prior to CLE treatment. For *in vitro* experiments, n represents the number of independent biological replicates (the separate passages of cells), with each replicate consisting of at least 3 technical replicates.

### Protein isolation

The old culture medium was discarded from cell culture dishes, and cells were washed three times with 1 ml of ice-cold PBS (Gibco). Lysis buffer (Solarbio) was supplemented with 50X phosphatase inhibitor mixture (Solarbio), 50X protease inhibitor mixture (Solarbio), and 0.05 M EDTA (Solarbio) in a 1:50 ratio and mixed well. After removing PBS, the lysis buffer was added to the dish on ice for 2–3 min for cell lysis. Cells were scraped off, transferred to a 1.5 ml centrifuge tube, mixed with lysate, and placed on ice for 5–10 min for lysis, with vortexing for 5 s every minute. After centrifugation at 13,000 rpm for 10 min at 4 °C, the supernatant (protein sample) was transferred to a new tube. Protein concentration was measured, an appropriate amount of sample buffer (Solarbio) was added proportionally, and the mixture was heated at 100°C for 5 min, cooled, and measured using a BCA protein assay kit (Solarbio). Samples were stored at –20°C.

### Cellular immunofluorescence

During passage, cells were seeded onto culture dishes containing pre-treated cover glasses. When cells reached near confluency, cover glasses were removed and washed twice with PBS (Gibco). Cells were fixed with 4% formaldehyde (Guangzhou Chemical Industry Group) for 10 min and permeabilized with 0.3% Triton X-100 in PBS for 10 min. Non-specific binding sites were blocked with 10% BSA (Beyotime) for 30 min. Cells were incubated with primary antibodies at 4 °C for 1 h or overnight: 1:1000 mouse LC3 monoclonal antibody (Cat No. 66139-3-Ig, Proteintech), 1:1000 LAMP1 rabbit recombinant antibody (Cat No. 98266-2-RR, Proteintech). Then, they were incubated with secondary antibodies for 30 min to 1 h: 1:1000 CoraLite^®^488-conjugated goat anti-mouse IgG (SA00013-1, Proteintech) or 1:1000 CoraLite^®^488-conjugated goat anti-rabbit IgG (SA00013-2, Proteintech). Washes with PBS were performed three times after each incubation. Nuclei were stained with DAPI for 10 min, followed by three PBS washes. Coverslips were mounted with anti-fade mounting medium onto glass slides and observed under a fluorescence microscope. All microscopy images were acquired using identical settings (laser power, exposure time, gain) across experimental groups for each fluorophore. Image analysis was performed using ImageJ with fixed threshold settings applied uniformly to all images from the same experiment to ensure consistency. For quantification, at least 10 cells per image were analyzed, and the average was calculated for each image. For immunofluorescence quantification in cell studies, three independent experiments were performed, each with 7 images per group (total n = 21 images per group).

### Grouping, modeling and administration of PD mice

Healthy male mice (8–10 weeks old, 22–25 g) were randomly assigned to MPTP+CLE (PD treatment), CTRL (NS/DMSO), or MPTP (chronic PD) groups (n = 14 per group). Male mice were used in this study to minimize hormonal variability, maintain consistency with previous MPTP model studies, and reflect the higher prevalence of PD in males (male:female ratio approximately 1.5:1). Mice were randomly assigned to experimental groups using a computer-generated randomization sequence. Sample size (n = 14 per group for behavioral studies, n = 7 per group for histology, n = 5 per group for protein analysis) was determined based on power analysis from pilot studies, with power = 0.8 and α = 0.05, to detect a 30% difference between groups. All behavioral tests, histological analyses, and Western blot quantifications were performed by investigators blinded to group allocation. Exclusion criteria were predefined: mice that died during the experiment (n = 0), failed to complete behavioral training (n = 0), or had technical issues during tissue collection (n = 0) were excluded. The MPTP+CLE group received MPTP+ [25 mg/kg/day, intraperitoneal injection (i.p.)] and CLE (10 mg/kg/day, i.p.; 1 mg/ml CLE in 10% DMSO/NS), with injection sites alternated daily. MPTP+ was administered every 3.5 days (twice weekly) for 18 times (approx. 2 months). CLE was administered daily for 16 times, with administration stopped 1 week before discontinuing MPTP+. The *in vivo* dose of 10 mg/kg/day was chosen based on established protocols showing CNS penetration and therapeutic efficacy in the mouse models of neurodegenerative diseases (Chen et al., 2021; Palma et al., 2022), as well as pharmacokinetic studies demonstrating that this dose achieves brain concentrations sufficient for target engagement. Treatment duration (16 doses over approximately 2 months) was designed to model the chronic exposure and allow the assessment of both motor and non-motor outcomes. The CTRL group received an equal volume of 10% DMSO/NS without CLE. The MPTP group received only MPTP+ as described. At the endpoint, mice were deeply anesthetized with sodium pentobarbital (50 mg/kg, i.p.). After the confirmation of consciousness loss, mice were subjected to the cardiac perfusion. Mice were euthanized by an overdose of sodium pentobarbital (150 mg/kg, i.p.) before the transcardial perfusion. For immunohistochemistry, mice were perfused with NS followed by 4% paraformaldehyde; for Western blot, only NS perfusion was used. For *in vivo* experiments, n = 14 mice per group were used for behavioral studies, n = 7 mice per group were used for tissue collection and immunohistochemistry, and n = 5 mice per group were used for protein analysis.

All animal studies and experiments were conducted in accordance with the Guide for the Care and Use of Laboratory Animals and were reviewed and approved by the ethics committee for animal care and use of Jiangxi Provincial People’s Hospital (The First Affiliated Hospital of Nanchang Medical College), China. All procedures in this study were conducted in accordance with the ethics committee for animal care and use of Jiangxi Provincial People’s Hospital (The First Affiliated Hospital of Nanchang Medical College), China (Ethic approval number: 2020-12-12).

### Rotarod test

As described previously (Shen et al., 2022), mice were trained on a rotarod apparatus in acceleration mode (4–40 rpm) and at a constant speed (16 rpm) until they could remain on the rod for at least 300 s. During testing, the rod accelerated from 4 to 40 rpm over 5 min, and the latency to fall was recorded. Due to transient sedation caused by CLE, its administration was stopped 1 week prior to behavioral testing.

### Fecal pellet analysis

After a 2-h fast, mice were placed individually in clean, transparent plastic cages. After 2 h, fecal pellets were collected, counted, and weighed for wet weight. Pellets were dried at 85 °C for 24 h and reweighed for dry weight. Moisture content percentage was calculated from the difference between wet and dry weights.

### Western blot

SDS-PAGE gels (8–12%; Bio-Rad) were prepared according to protein molecular weight. Samples (20 μg protein per lane) and 5 μl pre-stained marker were loaded. Electrophoresis was run in 1X buffer at 80 V for 30 min until the marker entered the separation gel, then at 100 V for ∼1 h until the dye front reached the bottom. Proteins were transferred to PVDF membranes at 30 V for 90 min. Membranes were blocked with 5% non-fat milk in PBST for 1 h and incubated with primary antibodies for 2 h at room temperature: 1:50,000 mouse GAPDH (60004-1-Ig, Proteintech), 1:2,000 mouse p-mTOR (Ser2448) (67778-1-Ig, Proteintech), 1:5,000 mouse t-mTOR (66888-1-Ig, Proteintech), 1:1000 rabbit p-AMPK (Thr183/172) (ab133448, Abcam), 1:1000 mouse p62 (66184-1-Ig, Proteintech), 1:1,000 rabbit LC3-I/II (orb411847, Biorbyt), 1:5,000 mouse P62 (66184-1-Ig, Proteintech), 1:500 mouse α-syn (66412-1-Ig, Proteintech). After washing, membranes were incubated with HRP-conjugated secondary antibodies: 1:1,000 goat anti-mouse (RGAM002, Proteintech) or 1:1,000 goat anti-rabbit (ab150077, Abcam) for 1–2 h at RT. Signals were detected using an enhanced chemiluminescence kit (Super ECL Plus S6009L; UElandy) as described (Li et al., 2022).

### Immunofluorescence staining

Brain tissues were fixed in 4% paraformaldehyde for 24 h, incubated in 20% sucrose in PBS (pH 7.5) until sunk (3–7 days), dried, embedded in OCT compound, and stored at −80 °C. Coronal sections (30 μm) were cut on a cryostat (Leica), collected on slides, permeabilized with 0.2% Triton X-100 for 5 min at RT, and blocked with 10% goat serum in PBS for 30 min at RT after rehydration. Sections were incubated with primary antibodies at 4°C overnight: 1:250 mouse LC3 (66139-3-Ig, Proteintech), 1:50 mouse TH (66334-1-Ig, Proteintech), 1:500 rabbit p-α-syn (S129) (ab51253, Abcam), 1:500 mouse α-syn (66412-1-Ig, Proteintech). After washing with PBST (0.2% Triton X-100), sections were incubated with secondary antibodies for 2 h at RT: 1:100 donkey anti-rabbit IgG (TRITC; ab6799, Abcam) or 1:100 goat anti-mouse IgG (A10680, Life Tech), conjugated to rhodamine (Red) or Alexa Fluor 488 (Green). After washing, nuclei were stained with DAPI (Blue; ab228549, Abcam) for 5 min at RT, washed, mounted with anti-fade medium, and imaged under a confocal microscope (Leica) with Photoshop. For tissue immunofluorescence, three mice per group were used, with 7 sections per mouse (total *n* = 21 images per group). We acknowledge that images from the same mouse are technical replicates and should not be considered independent biological replicates. Statistical analyses accounted for this by including mouse identity as a random effect where appropriate.

### Data analysis

Continuous data are expressed as mean ± SEM; categorical data as frequency or percentage. For normally distributed samples, independent sample *t*-test (two groups) or one-way ANOVA (multiple groups) was used. For non-normally distributed samples, Mann-Whitney U test (two groups) or Kruskal-Wallis test (multiple groups) was used. Correlation analysis used Spearman’s method: |r| > 0.9 (very strong), 0.7–0.9 (strong), 0.4–0.7 (moderate), ≤0.4 (weak/no correlation). *In vitro* experiments were repeated at least three times. *P <* 0.05 was considered statistically significant (*P < 0.05, **P < 0.01, ***P < 0.001).

## Results

### Identification of single-cell cell types

The cellular composition and the potential roles of different cell types in midbrain samples from 5 Parkinson’s disease (PD) patients and 6 healthy controls (HCs) were identified in dataset GSE157783. The dataset contained a total of 41,435 single cells, with 19,002 from PD patients (averaging approximately 3,800 cells per person) and 22,433 from HCs (averaging ∼3,739 cells per person). Based on cell-specific marker genes, twelve cell types were identified (Figure 2A): astrocytes, calcium-dependent activator protein for secretion 2 (CADPS2 or CAPS2) neurons, DaNs, endothelial cells, ependymal cells, excitatory neurons, gamma-aminobutyric acid neurons (GABAergic neurons), inhibitory neurons, microglia, oligodendrocytes, oligodendrocyte precursor cells (OPCs), and pericytes. The cells of the same type clustered together, with cluster size reflecting the proportion of each cell type. Significant differences were observed in the abundance of different cell types. Comparative clustering between the HC group (left, Figure 2B) and the PD group (right, Figure 2B) revealed differences in the proportional abundance of specific cell types, suggesting that PD onset might be associated with an imbalance in cellular composition.

**FIGURE 2:**
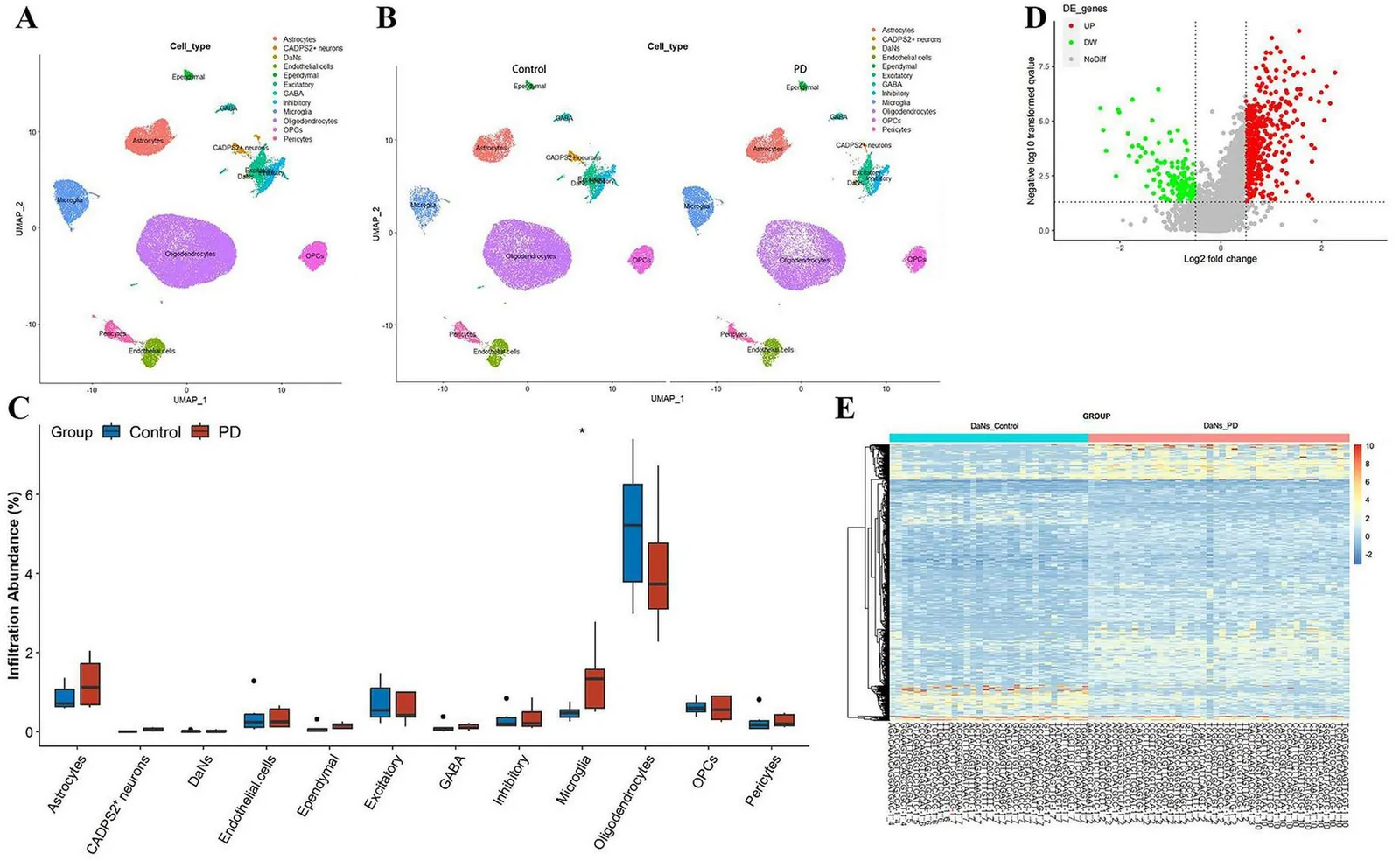
UMAP dimensionality reduction and cluster identification for cell type annotation. **(A)** UMAP plot identifying midbrain cell types, including astrocytes, CADPS2 neurons, dopaminergic neurons (DaNs), ependymal cells, excitatory neurons, GABAergic neurons, inhibitory neurons, microglia, oligodendrocytes, oligodendrocyte precursor cells (OPCs), and pericytes. Each point represents an individual cell, colored by cell type. **(B)** The distribution of substantia nigra (SN) cell types in healthy controls (HCs, left) and PD patients (right). **(C)** The relative proportions of cell types in the SN of PD patients and HCs. The proportion of microglia was significantly higher in PD patients (*P <* 0.05). The proportions of other cell types were not significantly different. Points in the box plot represent outliers. Mann–Whitney U test; *P <* 0.05. **(D)** Volcano plot displaying differential gene expression between PD and HCs. The x-axis shows log_2_ fold change; the y-axis shows the statistical significance (-log_10_ *P* value). Red dots: upregulated (UP) genes; green dots: downregulated (DOWN) genes; gray dots: non-significant (Nodiff) genes. **(E)** The Heatmap of differentially expressed genes in DaNs. Red indicates high expression; blue indicates low expression. Gene clusters are shown on the left. Sample names and group information (HCs, PD) are displayed at the bottom and top, respectively. UMAP, uniform manifold approximation and projection; CADPS2, calcium-dependent secretion activator 2; DaNs, dopaminergic neurons; GABA, gamma aminobutyric acid; OPCs, oligodendrocyte precursor cells; HCs, healthy controls; SN, substantia nigra.

### Cellular infiltration in the midbrain of PD patients

To identify potential cellular imbalances between HCs and PD patients, the Mann-Whitney U test was used to compare the proportions of different cell types. A box plot (Figure 2C) shows the distribution of cell type proportions in both groups. The proportion of microglia was significantly higher in the PD group compared to HCs (*P <* 0.05), while no significant differences were observed for other cell types (*P* > 0.05). These results suggest that microglia may play an important role in the pathophysiological processes of the PD midbrain.

### Differential gene expression analysis of DaNs

To explore the biological significance of gene expression differences between PD and HC groups, differential gene expression analysis was performed on the identified 74 DaNs. The using thresholds of |log_2_ fold change| > 0.5 and *P <* 0.05, 577 differentially expressed genes (DEGs) were identified, comprising 415 upregulated and 162 downregulated genes. A volcano plot (Figure 2D) provides a visual overview of these significant changes. Table 1 lists the top 20 upregulated and downregulated DEGs. This analysis provides a foundation for further functional and mechanistic studies.

**TABLE 1:** Top 20 differentially expressed genes (DEGs) (*P* < 0.001, |log_2_ FC| > 0.5).

Gene	log_2_ (fold change, FC)	Expression
FGF14	1.55	Upregulated
MALAT1	1.01	Upregulated
SCMH1	1.11	Upregulated
SYN2	1.18	Upregulated
NETO1	0.90	Upregulated
SCN1A	0.99	Upregulated
TRPC5	1.64	Upregulated
LRP4	0.88	Upregulated
CALD1	1.26	Upregulated
BAIAP3	0.84	Upregulated
CTC-4	−1.24	Downregulated
SV2C	−1.75	Downregulated
IL1RAPL2	−2.39	Downregulated
DCC	−2.03	Downregulated
ALDH1A1	−2.01	Downregulated
PLEKHA5	−1.36	Downregulated
PTPN13	−2.33	Downregulated
CATSPERG	−0.85	Downregulated
B4GALT6	−1.01	Downregulated
PDE4D	−1.84	Downregulated

### Cluster heatmap analysis of DEGs

The unsupervised hierarchical clustering of all DEGs was performed to reveal gene expression patterns. A heatmap was generated using the R Pheatmap package (Figure 2E). Compared to the HC group (green), DEGs were significantly upregulated in the PD group (red), indicating that the identified DEGs can effectively distinguish between the two groups. The clustering of DEGs suggests their potential involvement in distinct biological processes.

### GO functional enrichment analysis of DEGs

Gene Ontology (GO) enrichment analysis was performed to understand the biological processes associated with the upregulated and downregulated DEGs. Upregulated DEGs were primarily involved in nervous system-related processes, including synaptic function, membrane potential regulation, motor control, and axon formation (Figure 3A). Downregulated DEGs were enriched in processes related to neural signal transduction, such as ion channel activity, axon development, and neurotransmitter metabolism (Figure 3B). This analysis illustrates the distinct biological processes modulated by the DEGs.

**FIGURE 3:**
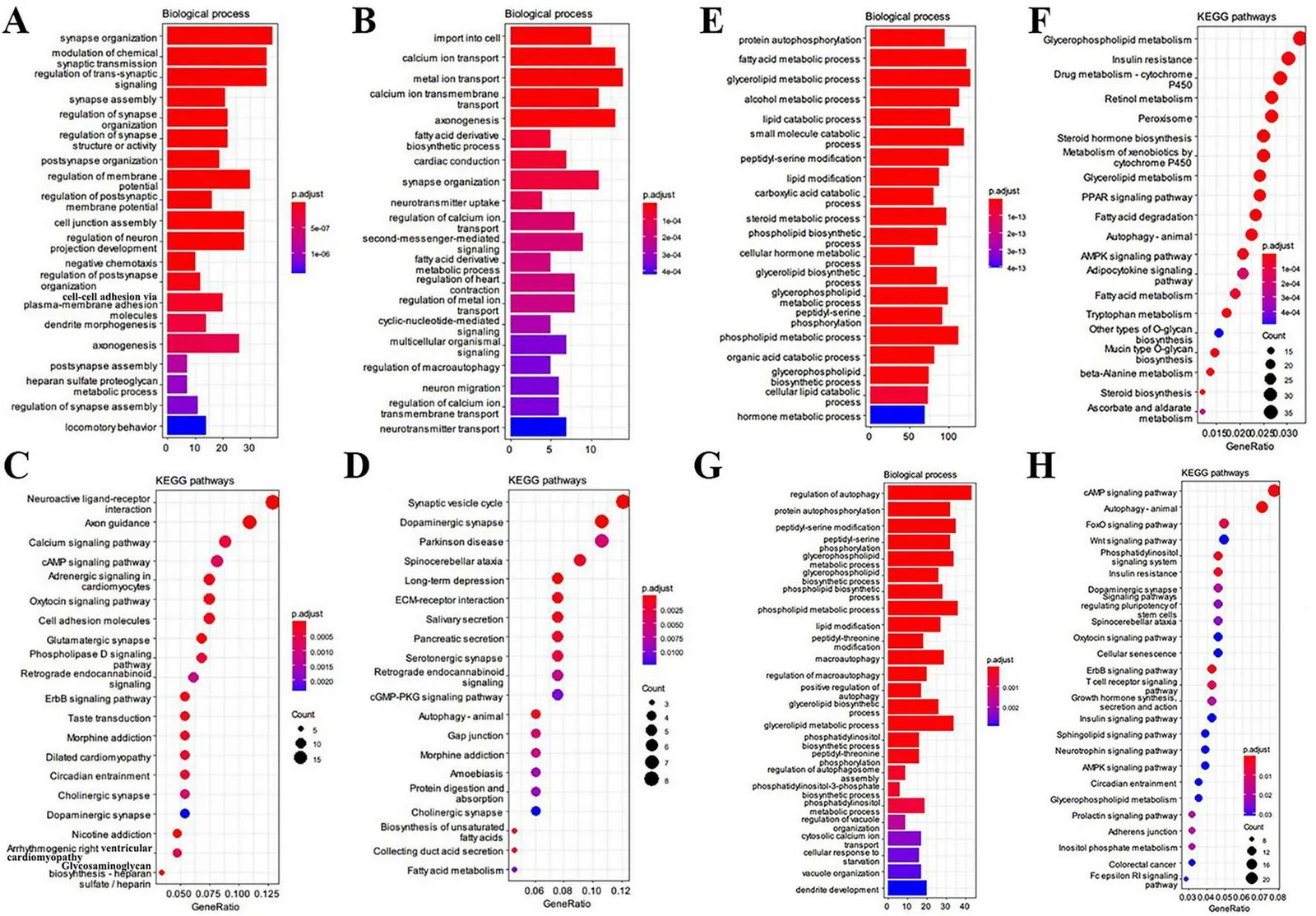
GO and KEGG pathway enrichment analysis of differentially expressed genes (DEGs). **(A)** The Bar chart of GO enrichment analysis for upregulated DEGs. **(B)** The Bar chart of GO enrichment analysis for downregulated DEGs. The y-axis shows enriched terms; the x-axis shows the number of genes. The color gradient represents the adjusted *P* value (p.adjust). **(C)** The Dot plot of KEGG pathway enrichment analysis for upregulated DEGs. **(D)** The Dot plot of KEGG pathway enrichment analysis for downregulated DEGs. The y-axis shows pathways; the x-axis shows the gene ratio. Dot size represents the number of genes; color represents the adjusted *P* value. **(E)** The GO enrichment analysis of molecular docking results. **(F)** The KEGG pathway enrichment analysis of molecular docking results. **(G)** The GO enrichment analysis of potential targets. **(H)** The KEGG pathway enrichment analysis of potential targets. GO, Gene Ontology; KEGG, Kyoto Encyclopedia of Genes and Genomes; DEGs, differentially expressed genes.

### KEGG pathway enrichment analysis of DEGs

KEGG pathway analysis was conducted to identify metabolic pathways involved in DEG regulation. Enrichment was evaluated based on GeneRatio, adjusted p-value (p.adjust), and the number of enriched genes. Upregulated DEGs were significantly enriched in nervous system pathways, including cholinergic, dopaminergic, and glutamatergic synapses, as well as axon guidance (Figure 3C). Downregulated DEGs were enriched in pathways related to cellular metabolism and neural signaling, such as autophagy, cholinergic synapses, and dopaminergic synapses (Figure 3D). The KEGG analysis highlights the key metabolic pathways associated with the DEGs.

### Correlation between braak staging and autophagy gene expression in the substantia nigra

Braak staging is an effective indicator of PD progression. Given the suggested dysregulation of autophagy pathways from GO and KEGG analyses, the relationship between Braak staging and autophagy gene expression in the substantia nigra was assessed using Spearman correlation. Thirty-eight autophagy genes showed moderate to strong correlation with Braak staging (|Spearman r| > 0.4, *P <* 0.05; Table 2). Genes with the strongest correlation included NFE2L2, ATG4C, MTMR14, and NFKB1, with NFE2L2 showing the highest correlation coefficient (r ≈ 0.77). This significant correlation suggests that autophagy dysfunction may be an important factor in PD progression.

**TABLE 2:** Correlation analysis between Braak staging of PD and autophagy genes in substantia nigra.

Autophagy gene	Spearman correlation	*P* value
NFE2L2	0.77	<0.001
ATG4C	0.75	<0.001
MTMR14	−0.74	<0.001
NFKB1	0.71	<0.001
NBR1	0.68	<0.001
KIF5B	0.66	<0.001
KLHL24	0.65	<0.001
PARP1	0.63	<0.001
BNIP1	−0.62	<0.001
RB1	0.61	0.001
ZFYVE1	−0.60	0.001
GRID1	−0.59	0.001
CAPN2	0.59	0.001
NPC1	0.58	0.001
HDAC1	0.58	0.001
ULK1	−0.57	0.002
CX3CL1	−0.55	0.002
MTOR	−0.53	0.004
CXCR4	0.52	0.004
FKBP1B	−0.52	0.004
ST13	0.51	0.005
ULK3	−0.51	0.006
PTK6	−0.51	0.006
MAPK9	−0.50	0.007
TM9SF1	−0.50	0.007
ATG9B	−0.50	0.007
PELP1	−0.49	0.008
SIRT1	0.48	0.009
ITPR1	−0.46	0.014
MAP2K7	0.45	0.017
CASP1	0.44	0.018
IKBKB	0.44	0.019
PTEN	−0.43	0.022
TP63	−0.43	0.024
SIRT2	0.42	0.027
ITGA3	−0.41	0.031
IKBKE	−0.41	0.032
DRAM1	0.41	0.034

### Molecular docking of CLE

Molecular docking was performed between the small molecule structure of CLE and 22,618 proteins. A total of 2,474 proteins with binding energies <−7 kcal/mol were identified, including 152 autophagy-related proteins. A binding energy <−7 kcal/mol is generally considered significant, with lower values indicating stronger binding. Table 3 lists the top 20 protein targets of CLE, and Table 4 lists the top 20 autophagy-related targets. Among them, the more detailed description of the autophagy-related targets identified through molecular docking, categorizing them based on their known functions: ULK1 and Beclin-1 proteins involve in autophagy initiation, ATG family proteins and LC3 Proteins involve in autophagosome formation and maturation, STX17 and VAMP8 proteins involve in autophagy-lysosome fusion, LAMP1 and LAMP2 proteins involved in lysosomal function, AMPK and mTOR are Key regulatory proteins (Supplementary Table 1).

**TABLE 3:** Top 20 proteins of molecular docking with CLE.

Proteins	Binding energy (kcal/mol)	Threshold
LSS	−11.7	<−7 kcal/mol
NT5M	−11.5	
FOLR3	−11.5	
PAQR5	−11.4	
DEGS1	−11.3	
SARDH	−11.1	
LMF2	−11.1	
TTPA	−10.9	
SEC14L6	−10.9	
LYRM7	−10.9	
LY86	−10.9	
LYRM4	−10.8	
DHCR7	−10.8	
TTC38	−10.7	
PKMYT1	−10.7	
DGKA	−10.7	
AKR1C3	−10.7	
SNRNP70	−10.6	
MT-CYB	−10.6	
MT-CO3	−10.6	

**TABLE 4:** The top 20 of autophagy related targets for molecular docking with CLE.

Proteins	Binding energy (kcal/mol)	Threshold
IKBKB	−10.4	<−7 kcal/mol
TM9SF1	−9.9	
ATG9A	−9.8	
PIK3C3	−9.7	
SPNS1	−9.6	
EIF2AK2	−9.6	
PTK6	−9.5	
ATG9B	−9.4	
P4HB	−9.3	
TNFSF10	−9.1	
DRAM1	−9.1	
MAPK9	−9.0	
GAA	−9.0	
BCL2L1	−9.0	
ULK2	−8.9	
RAF1	−8.9	
PRKCQ	−8.9	
MAPK8	−8.9	
EEF2	−8.9	
MTOR	−8.8	

### GO enrichment analysis of molecular docking results

GO enrichment analysis was performed on the top 20 protein targets from the molecular docking study. The enriched biological processes included protein phosphorylation, small molecule catabolic metabolism, fatty acid metabolism, and lipid catabolic metabolism (Figure 3E), indicating the key biological processes potentially modulated by CLE.

### KEGG pathway enrichment analysis of molecular docking results

The KEGG analysis of the top 20 protein targets revealed enrichment in pathways such as drug metabolism-cytochrome P450, PPAR signaling, fatty acid degradation, autophagy-animal, and AMPK signaling (Figure 3F), highlighting the metabolic pathways potentially involved in the CLE’s mechanism of action.

### Identification of CLE potential targets in PD and GO analysis

Intersecting the DEGs from DaNs with the CLE docking targets identified 167 potential targets of CLE in PD DaNs. GO analysis showed these targets were enriched in processes including protein autophosphorylation, macroautophagy, glycerolipid biosynthesis and metabolism, the positive regulation of autophagy, phosphatidylinositol biosynthesis, and the regulation of autophagosome assembly (Figure 3G).

### KEGG pathway enrichment analysis of potential targets

The KEGG analysis of the potential targets indicated enrichment in pathways such as cAMP signaling, autophagy-animal, dopaminergic synapse, neurotrophin signaling, and AMPK signaling (Figure 3H).

### CLE activates the AMPK/mTOR pathway in PC12 cells

To explore CLE’s effect on the AMPK/mTOR pathway, PC12 cells were treated and analyzed by western blot (WB) (Figure 4A). The p-AMPK/t-AMPK ratio was higher in the MPP^+^+CLE group than in the MPP^+^ group (*P <* 0.001). Conversely, the p-mTOR/t-mTOR ratio (activation) was significantly decreased in the MPP^+^, CLE, and MPP^+^+CLE groups (MPP^+^ vs. CTRL, *P* ≈ 0.035, CLE vs. CTRL, *P* ≈ 0.006, MPP^+^+CLE vs. CTRL, *P* ≈ 0.0012, MPP^+^+CLE vs. MPP^+^, *P* ≈ 0.047, respectively; Figure 4B) and was lower in the MPP^+^+CLE group than in the MPP^+^ group (*P <* 0.05). The ratio of p-AMPK/t-AMPK (activation) was significantly increased in the MPP^+^, CLE, and MPP^+^+CLE groups compared to CTRL (MPP^+^ vs. CTRL, *P* ≈ 0.0006, CLE vs. CTRL, *P* ≈ 0.00008, MPP^+^+CLE vs. CTRL, *P* ≈ 0.00002, PP^+^+CLE vs. MPP^+^, P ≈ 0.0018, respectively; Figure 4C). These results indicate that MPP^+^ and CLE increase AMPK phosphorylation and decrease mTOR phosphorylation at Ser2448 in PC12 cells, suggesting the activation of upstream AMPK signaling and reduced mTOR activity.

**FIGURE 4:**
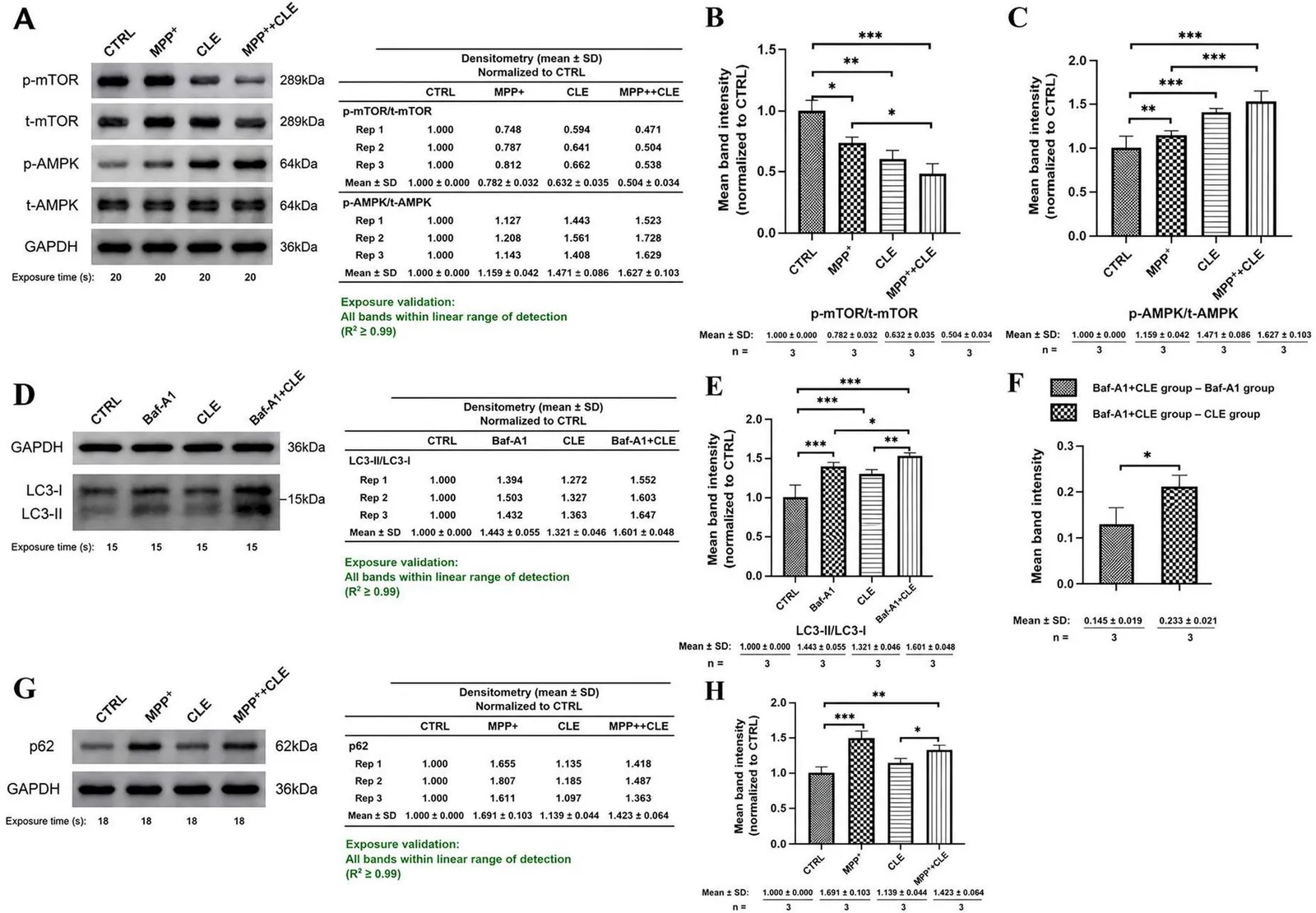
CLE increases AMPK phosphorylation, decreases mTOR phosphorylation at Ser2448, and enhances autophagy flux in PC12 cells. **(A)** The Western blot (WB) analysis of p-AMPK (Thr172), t-AMPK, p-mTOR (Ser2448), and t-mTOR protein levels in PC12 cells from the CTRL, MPP^+^, CLE, and MPP^+^+CLE groups. GAPDH is shown as a loading control. **(B)** The Quantification of p-mTOR/t-mTOR protein levels. **(C)** The Quantification of p-AMPK/t-AMPK protein levels. **(D)** The WB analysis of LC3-I and LC3-II expression. **(E)** The Quantification of the LC3-II/LC3-I ratio. **(F)** The Comparison of LC3-II/LC3-I levels in the Baf-A1, CLE, and Baf-A1+CLE groups, assessing autophagic flux. **(G)** The WB analysis of p62 expression. **(H)** The Quantification of p62 protein levels. Data are presented as mean ± SEM. Tukey’s multiple comparison test was performed after one-way ANOVA. *P < 0.05, **P < 0.01, ***P < 0.001; ns, not significant. CLE, clemastine; CTRL, control; WB, western blot; ANOVA, analysis of variance; SEM, standard error of the mean. n = 5 independent experiments.

### CLE activates autophagic flux in PC12 cells

To assess autophagic flux, LC3-I/II conversion was measured after treatment with Baf-A1, an autophagy inhibitor. Compared to the Baf-A1 group, the Baf-A1+CLE group showed a significantly increased LC3-II/LC3-I ratio (Baf-A1+CLE vs. Baf-A1, *P* ≈ 0.003; Figures 4D, E), indicating enhanced autophagosome formation. The difference in LC3-II/LC3-I between the Baf-A1+CLE and CLE groups was greater than that between the Baf-A1+CLE and Baf-A1 groups (Baf-A1+CLE-CLE vs. Baf-A1+CLE-Baf-A1; *P* ≈ 0.023; Figure 4F), suggesting CLE also promotes autophagosome degradation. Furthermore, CLE treatment (MPP^+^+CLE) significantly decreased the elevated p62 level induced by MPP^+^ (MPP^+^+CLE vs. MPP^+^, *P* ≈ 0.009; Figures 4G, H). As p62 degradation is negatively correlated with autophagy activity, these results suggest that CLE enhances autophagic flux by promoting both the formation and degradation of autophagosomes. Notably, while MPP^+^ activated upstream autophagy signaling (increased p-AMPK and decreased p-mTOR), the increased p62 levels and reduced lysosome numbers indicate impaired autophagic degradation, suggesting a state of autophagic flux blockade rather than functional autophagy completion.

### CLE increases lysosome number in MPP^+^-treated PC12 cells

Immunofluorescence staining for LC3 (autophagosomes), LAMP1 (lysosomes), and DAPI was performed (Figure 5A). MPP^+^ treatment significantly decreased lysosome number (*P <* 0.001 vs. CTRL), which was ameliorated by CLE co-treatment (*P <* 0.05; Figure 5B). CLE alone had no effect. The number of autophagosomes (LC3^+^LAMP1^+^) and autolysosomes (LC3^+^LAMP1^+^) increased significantly in all treatment groups compared to CTRL (Figures 5C, D). The MPP^+^+CLE group showed more autophagosomes and autolysosomes than the MPP^+^ group (*P <* 0.05 and *P <* 0.001, respectively). These results, combined with the WB data, suggest that CLE promotes autophagosome synthesis and enhances their fusion with lysosomes, thereby accelerating autophagic degradation.

**FIGURE 5:**
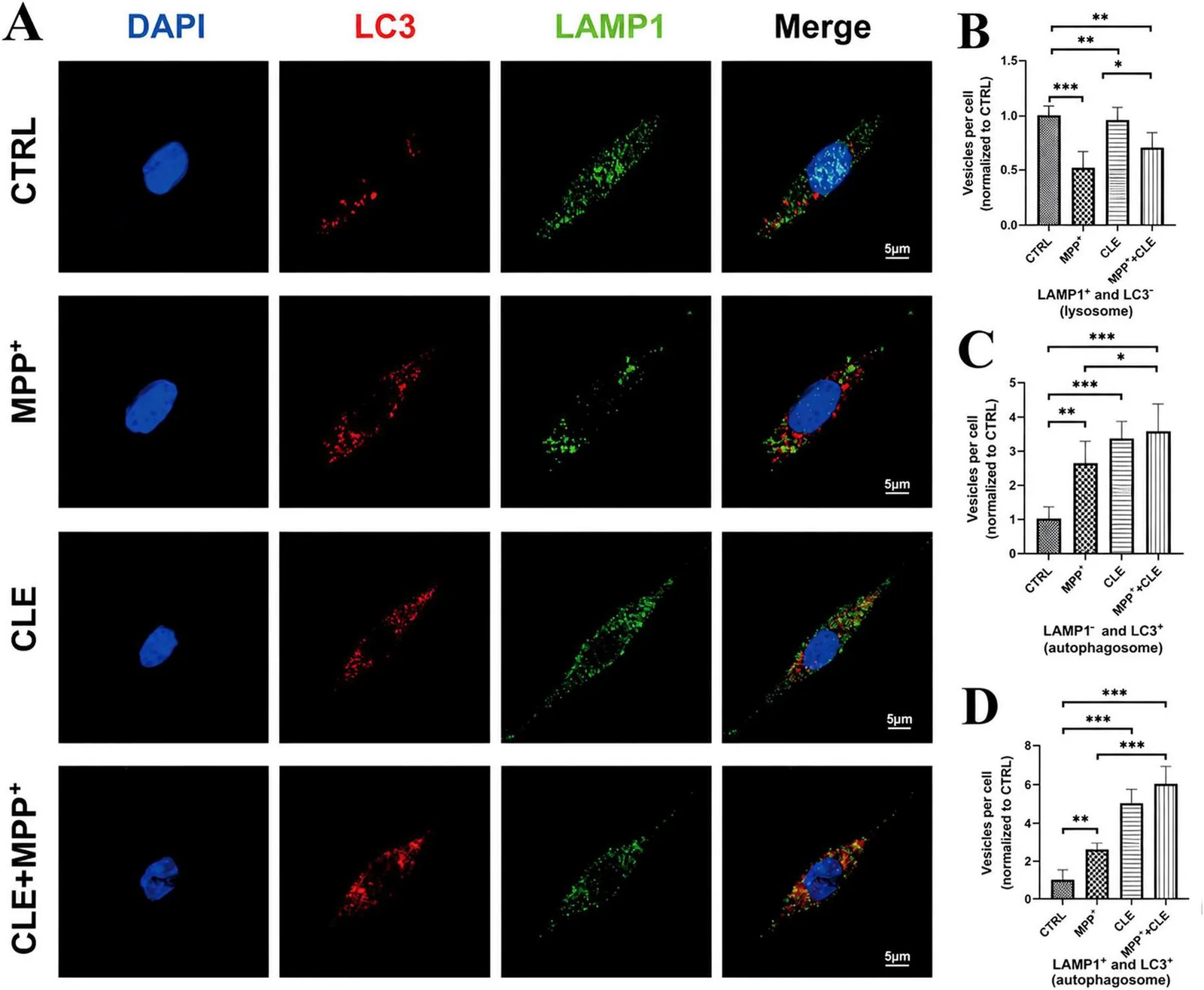
CLE improves autophagy-related organelles in PC12 cells. **(A)** Immunofluorescence staining for LC3 (red), LAMP1 (green), and DAPI (blue) in PC12 cells from CTRL, MPP^+^, CLE, and MPP^+^+CLE groups. Lysosomes are defined as LAMP1^+^LC3^–^ puncta; autophagosomes as LAMP1^+^LC3^+^ puncta; autolysosomes as LAMP1^+^LC3^+^ puncta. Scale bar: 5 μm. **(B)** The relative number of lysosomal puncta. **(C)** The relative number of autophagosome puncta. **(D)** The relative number of autolysosome puncta. Data are presented as mean ± SEM (N = 21 per group). Tukey’s multiple comparison test was performed after one-way ANOVA. *P < 0.05, **P < 0.01, ***P < 0.001; ns, not significant. CLE, clemastine; CTRL, control; ANOVA, analysis of variance; SEM, standard error of the mean. n = 21 images per group (7 images from 3 independent experiments).

### CLE is associated with reduced α-synuclein protein levels in MPP^+^-treated PC12 cells

WB analysis showed that MPP^+^ significantly increased α-synuclein (α-syn) levels (*P <* 0.001 vs. CTRL), which was associated with significantly reduced levels following CLE co-treatment (MPP^+^ vs. CTRL, *P* ≈ 0.00004; MPP^+^+CLE vs. MPP^+^, *P* ≈ 0.00009; CLE vs. CTRL, *P* ≈ 0.46; Figures 6A, B). CLE alone had no effect. CCK-8 assays showed that MPP^+^ decreased cell viability (MPP^+^ vs. CTRL, *P* ≈ 0.00002), which was significantly improved by CLE co-treatment (MPP^+^+CLE vs. MPP^+^, *P* ≈ 0.038; Figure 6C). CLE co-treatment modestly but significantly improved cell viability compared to MPP^+^ treatment alone (*P <* 0.05), suggesting partial protection against MPP^+^-induced toxicity.

**FIGURE 6:**
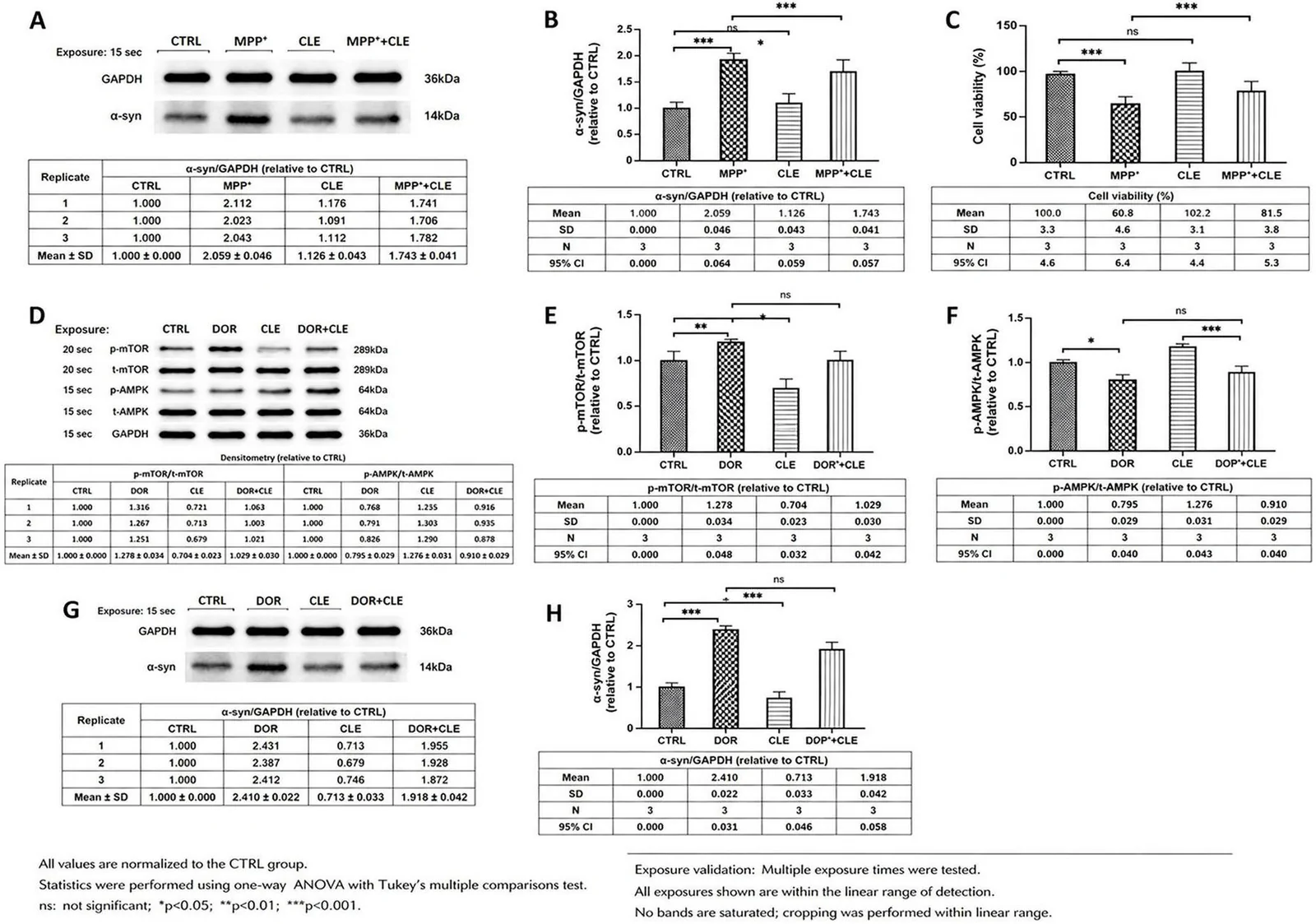
CLE reduces α-synuclein protein levels and improves cell survival, effects inhibited by dorsomorphin (DOR). **(A)** Western blot (WB) and **(B)** The quantification of α-synuclein (α-syn) protein levels in PC12 cells from CTRL, MPP^+^, CLE, and MPP^+^+CLE groups. GAPDH is shown as a loading control. **(C)** Cell survival rate in CTRL, MPP^+^, and MPP^+^+CLE groups. **(D, G)** The WB analysis of p-AMPK (Thr172), t-AMPK, p-mTOR (Ser2448), and t-mTOR protein levels in CTRL, DOR, CLE, and DOR+CLE groups. **(E, F, H)** The Quantification of the indicated protein ratios. Data are presented as mean ± SEM. Tukey’s multiple comparison test was performed after one-way ANOVA. *P < 0.05, **P < 0.01 ***P < 0.001; ns, not significant. CLE, clemastine; α-syn, α-synuclein; CTRL, control; WB, western blot; DOR, dorsomorphin; ANOVA, analysis of variance; SEM, standard error of the mean. n = 5 independent experiments.

### DOR inhibits the cLE-induced AMPK/mTOR signaling pathway in PC12 cells

The AMPK inhibitor Dorsomorphin (DOR) was used to investigate CLE’s target. DOR pretreatment effectively inhibited the CLE-induced increase in p-AMPK/t-AMPK and decrease in p-mTOR/t-mTOR (CLE vs. DOR+CLE, P ≈ 0.003; CLE vs. DOR+CLE, 0.00003; Figures 6D–F). Specifically, DOR reversed the effects of CLE on both p-AMPK/t-AMPK (*P <* 0.001 vs. CLE) and p-mTOR/t-mTOR (*P <* 0.01 vs. CLE). Furthermore, DOR cotreatment prevented the CLE-induced reduction of α-syn, resulting in higher α-syn levels in the DOR+CLE group compared to the CLE group (CLE vs. DOR+CLE, *P* ≈ 0.00015; Figures 6G, H). This is consistent with CLE’s effects being mediated through the AMPK/mTOR pathway.

### CLE activates the AMPK/mTOR pathway in the mouse midbrain

In a mouse PD model induced by MPTP, CLE treatment significantly increased the p-AMPK/t-AMPK ratio (MPTP+CLE vs. MPTP, *P* ≈ 0.006; Figures 7A, C) and further decreased the p-mTOR/t-mTOR ratio (MPTP+CLE vs. MPTP, P ≈ 0.014; Figures 7A, B) in the substantia nigra, confirming pathway activation *in vivo*.

**FIGURE 7:**
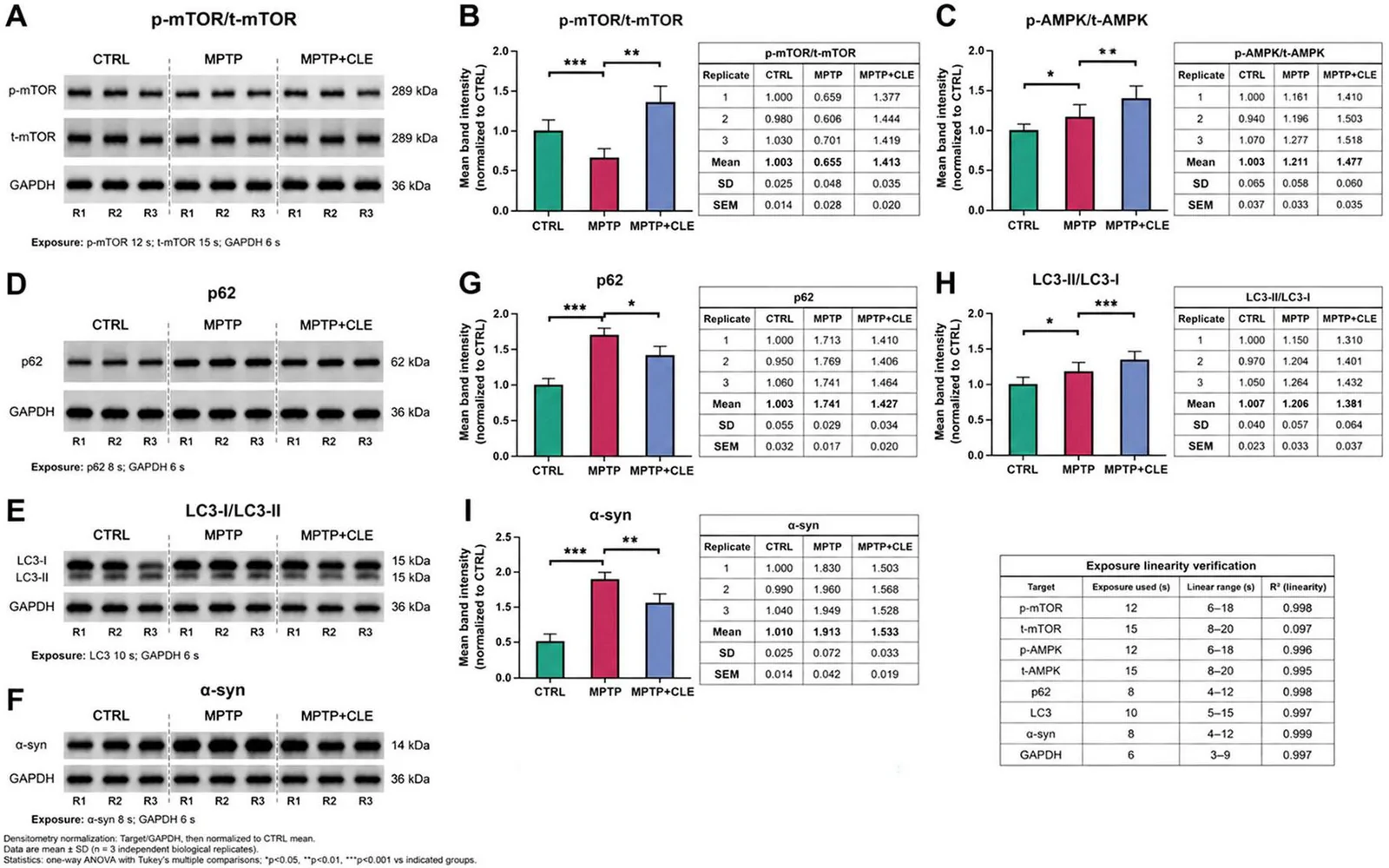
CLE increases AMPK phosphorylation, decreases mTOR phosphorylation at Ser2448, enhances autophagy, and reduces α-synuclein levels in the mouse midbrain. **(A)** The WB analysis of p-AMPK (Thr172), t-AMPK, p-mTOR (Ser2448), and t-mTOR protein levels in the midbrain of mice from CTRL, MPTP, and MPTP+CLE groups. GAPDH is shown as a loading control. **(B)** The quantification of p-mTOR/t-mTOR levels. **(C)** The quantification of p-AMPK/t-AMPK levels. **(D–F)** The WB analysis of p62, LC3-I/II, and α-syn protein levels. **(G–I)** The quantification of p62, LC3-II/LC3-I ratio, and α-syn protein levels. Data are presented as mean ± SEM. Tukey’s multiple comparison test was performed after one-way ANOVA. *P < 0.05, **P < 0.01, ***P < 0.001. CLE, clemastine; α-syn, α-synuclein; CTRL, control; WB, western blot; ANOVA, analysis of variance; SEM, standard error of the mean. n = 5 independent experiments.

### CLE enhances autophagy and reduces α-syn in the mouse midbrain

WB analysis showed that CLE treatment increased the LC3-II/LC3-I ratio (MPTP+CLE vs. MPTP, *P* ≈ 0.0025; Figures 7E, H) and decreased p62 levels (MPTP+CLE vs. MPTP, *P* ≈ 0.012; Figures 7D, G) in the substantia nigra, indicating enhanced autophagy. Concurrently, CLE significantly reduced the MPTP-induced elevation of α-syn protein levels (MPTP+CLE vs. MPTP, *P* ≈ 0.009; Figures 7F, I).

### CLE increases LC3 in DaNs and reduces p-α-syn in mice

Immunofluorescence staining confirmed that CLE treatment increased LC3 puncta in tyrosine hydroxylase-positive (TH^+^) DaNs (MPTP+CLE vs. MPTP, *P* ≈ 0.0005; Figures 8A, B). Furthermore, CLE significantly reduced the levels of phosphorylated α-syn (Ser129) in DaNs (MPTP+CLE vs. MPTP, *P* ≈ 0.0005; Figures 8C, D).

**FIGURE 8:**
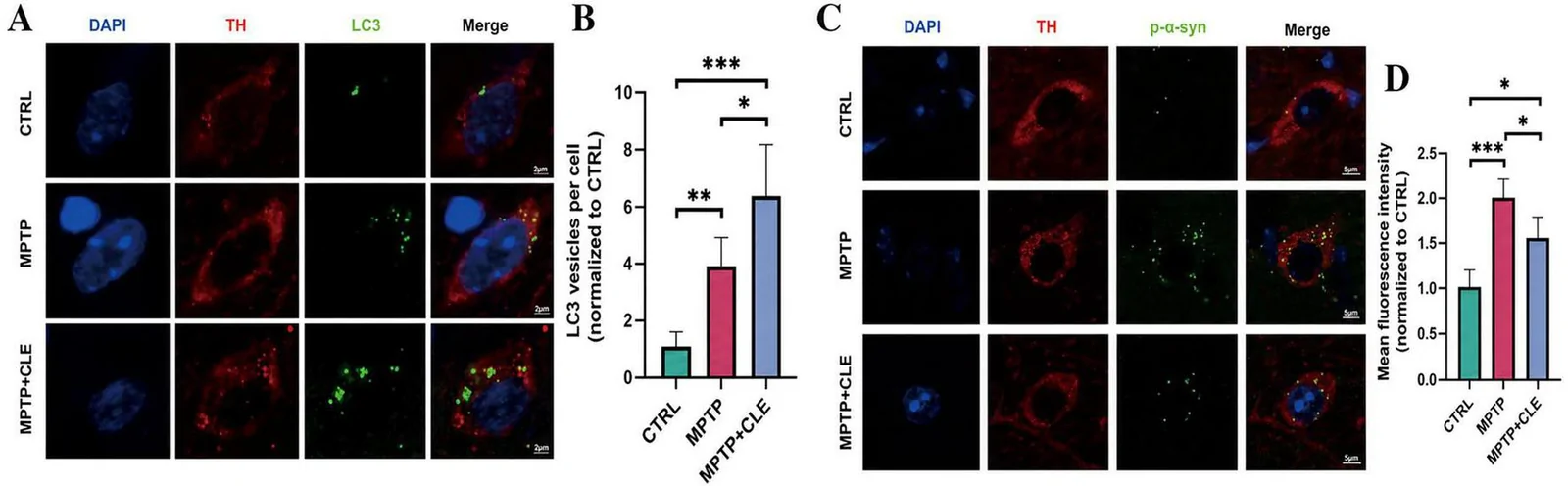
CLE increases LC3 and reduces phosphorylated α-synuclein in dopaminergic neurons (DaNs) of MPTP-treated mice. **(A)** Immunofluorescence staining for TH (red, marker for DaNs), LC3 (green), and DAPI (blue) in the substantia nigra (SN) of mice from CTRL, MPTP, and MPTP+CLE groups. Scale bar: 2 μm. **(B)** The quantification of the relative number of LC3 puncta in DaNs. **(C)** Immunofluorescence staining for TH (red), p-α-syn (Ser129, green), and DAPI (blue) in the SN. Scale bar: 5 μm. **(D)** The quantification of the relative fluorescence intensity of p-α-syn (Ser129) in DaNs. Data are presented as mean ± SEM (N = 21 per group). Tukey’s multiple comparison test was performed after one-way ANOVA. *P < 0.05, **P < 0.01, ***P < 0.001. CLE, clemastine; DaNs, dopaminergic neurons; p-α-syn, phosphorylated α-synuclein (Ser129); SN, substantia nigra; CTRL, control; ANOVA, analysis of variance; SEM, standard error of the mean. n = 21 images per group (7 images from 3 mice per group).

### CLE protects DaNs and ameliorates motor and non-motor symptoms in mice

CLE treatment significantly attenuated the MPTP-induced loss of TH^+^ DaNs in the substantia nigra (MPTP+CLE vs. MPTP, *P* ≈ 0.012; Figures 9A, B). In behavioral tests, CLE improved the latency to fall on the rotarod (MPTP+CLE vs. MPTP, *P* ≈ 0.003; Figure 9C), indicating improved motor coordination. CLE also ameliorated constipation-like symptoms, increasing both fecal pellet count (MPTP+CLE vs. MPTP, *P* ≈ 0.014; Figure 9D) and fecal water content (MPTP+CLE vs. MPTP, *P* ≈ 0.0018; Figure 9E). CLE significantly increased TH^+^ DaNs survival in the substantia nigra (Figures 9A, B). CLE improved both motor function (rotarod test, Figure 9C) and non-motor symptoms (fecal parameters, Figures 9D–E). These *in vivo* improvements suggest the functional neuroprotection despite the modest *in vitro* cell viability effect.

**FIGURE 9:**
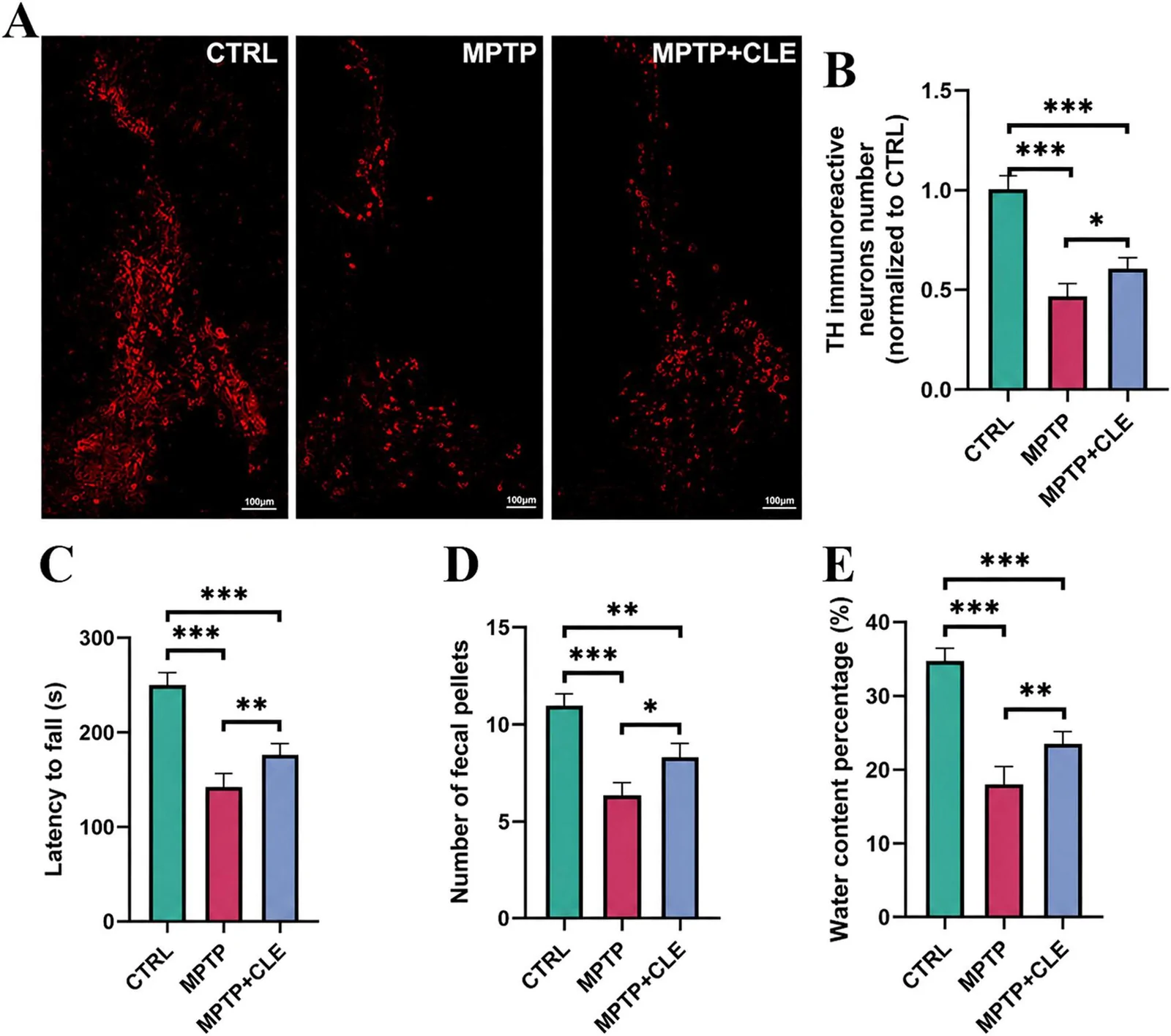
CLE improves the number of dopaminergic neurons and behavior in MPTP-treated mice. **(A)** The representative images and **(B)** The quantification of TH-positive dopaminergic neurons (DaNs) in the substantia nigra (SN) of mice from CTRL, MPTP, and MPTP+CLE groups. Scale bar: 100 μm (N = 7 per group). **(C)** Latency to fall in the rotarod test. **(D)** Total number of fecal pellets produced per mouse over 2 h. **(E)** Fecal water content (N = 14 per group). Data are presented as mean ± SEM. Tukey’s multiple comparison test was performed after one-way ANOVA. *P < 0.05, **P < 0.01, ***P < 0.001. CLE, clemastine; DaNs, dopaminergic neurons; SN, substantia nigra; CTRL, control; ANOVA, analysis of variance; SEM, standard error of the mean. *n* = 14 per group for behavior, *n* = 7 per group for histology.

## Discussion

Single-cell sequencing is a sequencing technology that can obtain the genetic information from the individual cells. It can be used in studying the genome or transcriptome of individual cells, revealing heterogeneity and function between cells. However, due to the high cost of single-cell sequencing and the scarcity of human midbrain substantia nigra specimens, the present study on the single-cell sequencing of the midbrain is very inefficient, this study only analyzed the single-cell transcriptome data from 5 PD cases and 6 age-matched HCs of the midbrain after death. The results found that a total of 12 cell types were identified from 41,435 single cells obtained from the midbrain (Figure 2A), including astrocytes, CADPS2 neurons, DaNs, endothelial cells, ependymal cells, excitatory neurons, gamma aminobutyric acid neurons, inhibitory neurons, microglia, oligodendrocytes, oligodendrocyte precursor cells and pericytes. These cell types are mainly composed of neurons, glial cells and vascular cells, consistent with previous studies (Chen et al., 2006).

In Figure 2B, it can be seen that oligodendrocytes are the most abundant cell type in the midbrain. In this study, compared to HCs group, the PD group showed a slight decrease in oligodendrocytes, but there was no statistically significant difference. The main function of oligodendrocytes is to form myelin sheaths on neuronal axons, and they can also participate in the immune function of the nervous system, clearing harmful substances and repairing damage. The role of oligodendrocytes in PD is not yet very clear, but they may be related to the formation and evolution of PD. For example, in PD, oligodendrocytes may be damaged by α-syn, resulting in demyelination and neuronal degeneration in the pathogenesis of PD (Ding et al., 2020).

Among them, some genetic variations are associated with oligodendrocyte dysfunction and an increased risk of PD, such as GBA, LRRK2, SNCA, etc. (Tulisiak et al., 2019; Choudhury et al., 2022). Oligodendrocytes may be a potential target for PD treatment by promoting myelin repair or clearing α-syn to protect neurons (Custodia et al., 2021). DaNs are the most studied cell type in PD. However, it was suggested in Figure 2C that compared with HCs group, there was no statistical change in the number of DaNs in the PD group. The reason might be that the substantia nigra was not specifically sampled, resulting in a relatively small number of DaNs obtained this time, accounting for only 0.18% of total cell count, thereby limiting the statistical results of the number of DaNs. Although there was no statistical difference in quantity, it is still meaningful for us to conduct relevant analyses on the intrinsic gene expression levels of DaNs later.

In addition, it was suggested in Figure 2C that compared with HCs group, the number of microglia in the PD group increased significantly. The role of microglia as immune cells in the nervous system is rather complex. On the one hand, they can engulf and clear excessive α-syn which the main pathological marker of PD is (Liu et al., 2023). On the other hand, microglia also release inflammation factors, the roles of these inflammatory factors may lead to the neuronal death and neurodegeneration (Jimenez et al., 2008). The impact of microglial proliferation also depends on their activation status and function (Willis et al., 2020), which is mainly divided into two states: the classical activation type (M1 phenotype) and the substitution activation type (M2 phenotype). The transformation of this state is called the polarization process (Wan et al., 2016). In PD, the polarization state of microglia may have a significant impact on the survival and death of neurons. The microglia of M1 phenotype exacerbate nerve injury because they mainly release inflammatory factors (Xiang et al., 2018). The microglia of M2 phenotype play an important role in alleviating nerve injury and promoting nerve repair because they mainly release anti-inflammatory factors (Xiang et al., 2018). Since single-cell sequencing is currently difficult to accurately distinguish M1 and M2 subtypes, further research on the related subtypes of microglia has been limited.

In Figure 2C, the number of astrocytes between two groups is not obvious change. Astrocytes are a type of glial cells present in both brain and spinal cord. They have multiple functions, such as assisting, guiding and nourishing nerve cells, maintaining the ionic stability of neurons, and participating in the formation of the blood-brain barrier, etc. They can also proliferate and repair tissues when the central nervous system is damaged. In PD, the roles of astrocytes are complex. They can both protect neurons and exacerbate neurodegeneration. The roles of astrocytes mainly depend on their activation status, signaling pathways and gene mutations (Marchetti, 2018). The present study has shown that some gene mutations in astrocytes, such as PINK1, PRKN and LRRK2, may lead to the phenotype of PD and affect the functions of astrocytes (Wang et al., 2021). In addition, it was also found that astrocytes can promote the occurrence of neuroinflammation because they regulated the NF-κB signaling pathway to respond to inflammatory signals (Gaojian et al., 2020), while the dopamine receptor Drd2-β -Arrestin2 signaling pathway in astrocytes can also inhibit neuronal death and neuroinflammation in the pathogenesis of PD.

In the DaNs of the substantia nigra, DEGs between HCs group and the PD group were analyzed to reflect the changes in the biological process of PD and to attempt to identify potential markers for disease diagnosis or treatment. The results presented in Figure 3C demonstrate that, compared with HCs group, most of the differentially upregulated genes in the PD group were related to synapses. In fact, PD is closely related to synapses. DaNs form synaptic connections with neurons in the striatum through axons (Ge et al., 2020). The α-syn protein is expressed in the presynaptic and perinuclear regions of DaNs and is involved in regulating the release and reuptake of neurotransmitters such as dopamine. In PD, the Lewy body (LB) formed by the misfolding and aggregation of α-syn can impair DaNs and the synaptic function between them and spiker neurons in the striatum, resulting in a decrease in dopamine levels and thereby causing motor disorders. Therefore, PD not only affects the survival of individual neurons, but also influences the stability and plasticity of the entire synaptic tissue (Custodia et al., 2021).

The results presented in Figure 3C demonstrate that the upregulated DEGs are mainly enriched in various different signaling pathways. For example, the specific role of neuroactive ligand-receptor interactions in PD may be related to dopamine receptor signal transmission and neuroinflammation, such as the loop mechanism (Yang et al., 2019). Axonal guidance refers to the mechanism by which the axons of neurons find the correct target cells during their development. Axonal guidance pathway genes are genes involved in regulating axonal guidance, and they are crucial for the formation and function of the nervous system. The axons of DaNs need to be maintained, regenerated and target recognized through axon steering pathway genes (Asghari Adib et al., 2018). The relationship between the calcium signaling pathway and PD is also closely related. For instance, calcium-independent phospholipase A2β is an enzyme involved in the repair of oxidized phospholipids. Its mutation can lead to an imbalance in calcium ion homeostasis, thereby causing the mitochondrial dysfunction, iron metabolism disorders and the neuronal death (Sun et al., 2021). Voltage-gated calcium ion channels are a type of channel that controls the entry of calcium ions into cells, and they regulate the release of neurotransmitters on the presynaptic membrane. The abnormal expression or function of voltage-gated calcium ion channels may affect the activity and survival of DaNs, thereby being associated with the pathogenesis of PD (Dolphin and Lee, 2020).

In PD, some genes with downregulated expression levels may play a role in protecting neurons. The gene downregulation indicates that the corresponding enriched biological process or signaling pathway is impaired (Figure 3B). For instance, the present study found that the disorder of metal ion transport in PD patients leads to the imbalance of the homeostasis of biometal ions, such as iron, copper, zinc, etc., and the accumulation of heavy metals, such as manganese, lead, mercury, etc., and metal ions may cause α-syn misfolding and accelerate α-syn aggregation by binding α-syn, thereby forming LB. It is worth noting that neurotransmitter uptake dysfunction has a huge impact in the pathogenesis of PD. 5-hydroxytryptaminergic neurons can take up the exogenous levodopa, synthesize dopamine, and release it as a pseudo-neurotransmitter. Due to the lack of effective reuptake ability, the extracellular dopamine concentration fluctuates (Fridjonsdottir et al., 2021). In addition, the glutaminergic synaptic input is also involved in the formation and regulation of PD-related electroencephalogram rhythms and tremor phenomena. Fatty acids are important biomolecules and play a certain role in the pathogenesis of PD. For instance, a special fatty acid phospholipase iPLA2β can hydrolyze long-chain lipids and eliminate the signal of ferroptosis, thereby reducing the neuronal death and movement disorders in PD.

Furthermore, there are also varying the degrees of disorders in the signaling pathways. The results presented in Figure 3D showed that dopaminergic synaptic disorders might involve α-syn stability. Specifically, α-syn, as a highly enriched small molecule protein in neurons, can regulate the formation, transport and release of synaptic vesicles. In PD, α-syn undergoes the abnormal aggregation and denaturation, forming the insoluble LB, which can impair the normal function of DaNs and induce apoptosis (Hallacli et al., 2022). Furthermore, autophagy has also been impaired to varying degrees. The role of autophagy in PD is multi-faceted. Autophagy can degrade α-syn through the lysosomal pathway or the non-lysosomal pathway, thereby reducing its aggregation and toxicity (Cogo et al., 2020). Autophagy can clear the damaged or dysfunctional mitochondria, thereby protecting neurons from oxidative stress and energy crises (Sliter et al., 2018; Cogo et al., 2020). Autophagy can regulate other PD-related signaling pathways within neurons, such as NF-κB, NLRP3 inflammasome, mTOR, etc. (Chen et al., 2022). Autophagy may be an effective neuroprotective agent or therapeutic strategy, as inducing or enhancing autophagy levels can delay or improve neuronal damage and dysfunction in PD model animals or cell models.

The Braak staging of PD is a model that divides the pathological progression of PD into six stages based on the distribution and spread of LB in the nervous system. The basic idea is that LB in PD first appears in the peripheral nervous system and the olfactory system, and then gradually spreads upward to the brainstem, midbrain, the limbic system and the cerebral cortex. Braak staging can help us understand the pathological mechanism of PD, explore the diffusion pattern and the influencing factors of LB in the nervous system, assist us in evaluating the severity and prognosis of PD, guide the individualized treatment plans and follow-up plans, and provide the theoretical basis and the experimental models for finding new biomarkers, drug targets and treatment strategies. In the differential gene analysis of DaNs, it was found that autophagy disorder exists in PD. To explore the correlation between autophagy and the severity of PD, a correlation analysis between autophagy genes and Braak staging was conducted. The data presented in Table 2 demonstrate that many autophagy-related genes are correlated with Braak staging, indicating that autophagy may be an important factor affecting Braak staging. Molecular docking is an important method for structure-based drug designing and screening, which can predict and evaluate the affinity, binding mode and binding energy between molecules by studying the interaction between ligand molecules and receptor molecules. Molecular docking was conducted with 22,618 proteins using the small molecule structure data of CLE. There were 2,474 interacting proteins with binding energy less than -7 kcal/mol. Top 20 results were presented in Table 3, among which there were 152 autophagy-related proteins. Top 20 results were presented in Table 4. These results suggested that CLE might be able to regulate the autophagy process in various diseases. Relevant literature concluded through mass spectrometry analysis that CLE could effectively pass through the blood-brain barrier (Turski et al., 2018). Based on these results, it is speculated that CLE may have a regulatory effect on the autophagy process of the central nervous system. To explore the biological function of CLE in PD, the possible targets of CLE were intersected with the DEGs of DaNs in PD as the potential targets of CLE in PD. Make use of potential targets, GO and KEGG enrichment analyses were performed to obtain the relevant biological process Figure 3E and the signaling pathway information, suggesting a connection with the processes or pathways related to autophagy. Among the 152 autophagy-related proteins identified through molecular docking, many are known to function at specific steps in the autophagic pathway. The molecular docking results, combined with the enrichment of autophagy-related pathways in GO and KEGG analyses, guided our focused investigation of the AMPK/mTOR pathway as a potential mechanistic target of CLE in PD. The AMPK/mTOR axis represents a particularly significant finding, as these proteins serve as the master regulators of autophagy. AMPK directly phosphorylates ULK1 to initiate autophagy, while mTORC1 negatively regulates autophagy through the phosphorylation of ULK1 and ATG13. The identification of both upstream regulators (AMPK, mTOR) and downstream effectors (ATG proteins, LC3, LAMP1) suggests that CLE may act at multiple levels of the autophagy pathway.

The death of DaNs in the substantia nigra and the formation of LB are the main pathological features of PD (McNaught and Olanow, 2003). LB is formed by α-syn accumulated in the cytoplasm, which indicates that α-syn is a crucial factor in the pathogenesis of PD (Chen et al., 2019). Autophagy can eliminate harmful proteins and organelles within cells. In PD, the disorder of the autophagy pathway is also associated with the occurrence and progression of the disease. By activating the autophagy pathway, it helps to enhance the clearance and degradation of α-syn, thereby protecting neurons from damage (Choi et al., 2018). Therefore, the study on interaction between α-syn and the autophagy pathway is to help us better understand the pathogenesis of PD and provide guidance for finding new treatment methods. MPTP is a dopaminergic neurotoxin that can cause symptoms similar to those of PD by destroying DaNs that produce dopamine in the substantia nigra. Therefore, it is widely used in the research of various animal models of PD. PC12 cells are derived from pheochromocytoma in the adrenal medulla of rats and possess the characteristics of neuroendocrine cells, capable of secreting neurotransmitters such as dopamine and norepinephrine. PC12 cells are also widely used in the neurology research, such as neurotoxicity, neuroprotection, neurosecretion, neuroinflammation and synaptogenesis. MPP^+^ is a metabolite of MPTP and also is a neurotoxin. It can interfere with the oxidative phosphorylation by inhibiting the activity of mitochondrial complex I, leading to ATP depletion and cell death (Jethva et al., 2011). MPP^+^ induced PC12 cells are often used as the cell model of PD.

AMPK protein plays an important role in the cellular metabolic regulation and is a key energy metabolism regulatory protein. Autophagy is regulated by multiple signaling molecules, one of which is AMPK, which can activate the autophagy initiation complex ULK1 by inhibiting the mTORC1 signaling pathway, and promote the formation and maturation of autophagy vesicles by activating other signaling molecules such as Beclin-1, VPS34, etc. (Yan et al., 2019). There exists a mutual influence relationship between AMPK and α-Syn, and it may involve different types, the mutations and modified states of α-Syn. α-Syn can inhibit AMPK activity or downstream effects through different mechanisms, thereby reducing the level of autophagy (Rocha et al., 2018). In the PD model, activating or enhancing AMPK reduces the autophagic flow obstruction induced by α-Syn through inhibiting mTORC1, promotes the metabolism and clearance of α-Syn, and thereby improves its pathogenicity (Yuan et al., 2022). In addition, it is also found that the activation of AMPK protein can inhibit the aggregation and toxicity of α-syn, thereby protecting neurons from damage (Paudel et al., 2020).

mTOR belongs to the PI3K-associated kinase family and is a serine/threonine kinase that can bind to various proteins. Two different complexes are formed, namely mTORC1 and mTORC2. The signal transduction of mTORC1 and mTORC2 is crucial for the regulation of autophagy. Among them, mTORC1 has a close signal interaction with ULK1, while mTORC2 is related to the transport of autophagosomes. mTOR is the main regulatory molecule for cell growth and metabolism. Activating mTOR can stimulate the anabolic processes while inhibiting catabolic processes, such as autophagy. The phosphorylation status of mTOR at Ser2448 is commonly used as an indicator of mTORC1 activity, though mTOR inhibitors, as the main regulators of autophagy, some mTOR inhibitors have been approved for the clinical treatment, and some have shown beneficial effects in anti-aging and prolonging life. Phosphorylation is a common form of post-translational modification in the process of cell signal transduction. p-AMPK is the phosphorylated AMPK and t-AMPK is total AMPK. The ratio of p-AMPK/t-AMPK can reflect the activation degree of AMPK. Similarly, p-mTOR is the phosphorylated mTOR and t-mTOR is total mTOR. The ratio of p-mTOR/t-mTOR can reflect the activation degree of mTOR. Figure 4C demonstrates that, on PC12 cells, compared with the CTRL group, the phosphorylation expression levels at the Thr183 and Thr172 sites on the α subunit of AMPK protein in the MPP^+^ group were higher. The activation of AMPK involves the phosphorylation of the Thr172 and Thr183 sites on the α subunit, and this process is influenced by the levels of AMP and ATP. When adenosine monophosphate (AMP) to adenosine triphosphate (ATP) ratio in cells increases, AMPK is activated, which usually occurs under the conditions of cell stress or energy consumption (Paudel et al., 2020). Our experimental results indicate that MPP^+^ can increase the phosphorylation of AMPK (Figure 4C). It is worth noting that MPP^+^ is a neurotoxin that selectively destroys DaNs in the substantia nigra of brain, thereby leading to the symptoms of PD. The role of AMPK in PD may be related to regulating autophagy, protecting neurons and improving the intestinal barrier, etc. It has found that MPP^+^ increases the ratio of AMP to ATP by inhibiting the complex I of the mitochondrial electron transport chain, resulting in a decrease in ATP production. The increase in AMP level (Risiglione et al., 2020) further activates AMPK, thereby promoting energy protection and production in response to cellular stress. Therefore, the activation of AMPK might be a stress response of PC12 cells in the MPP^+^ group. However, the toxicity of MPP^+^ eventually led to cell apoptosis. The observation that MPP^+^ increases AMPK phosphorylation and decreases mTOR phosphorylation while simultaneously causing p62 accumulation may appear contradictory. However, this apparent contradiction can be explained by the concept of autophagic flux impairment. MPP^+^-induced AMPK activation and mTOR inhibition represent an adaptive stress response – the cell attempts to upregulate autophagy in response to mitochondrial dysfunction and energy depletion. However, this signaling response alone does not guarantee functional autophagy completion. As shown in Figure 5B, MPP^+^ significantly reduces lysosome numbers, impairing the terminal step of autophagy. This creates a ‘bottleneck’ where autophagosomes are formed but cannot be efficiently degraded, leading to autophagic flux blockade. The increased p62 levels directly reflect this impaired degradation, which is consistent with the downregulation of autophagy-related genes observed in PD patients. Thus, MPP^+^ triggers autophagy signaling (AMPK phosphorylation, mTOR inhibition) but the downstream execution is compromised due to lysosomal dysfunction, representing a state of incomplete or ineffective autophagy – a common feature in neurodegenerative diseases.

The results presented in Figure 4B showed that, compared with the CTRL group, the phosphorylation expression level of the mTOR protein at the Ser2448 site in the MPP^+^ group was lower. mTOR Ser2448 is an important phosphorylation site that can be regulated by upstream proteins such as AMPK and PI3K. The phosphorylation of mTOR Ser2448 enhances the kinase activity of mTOR and inhibits autophagy through downstream pathways (Tao et al., 2021). This suggests that the MPP^+^ group may have promoted autophagy by reducing mTOR phosphorylation at Ser2448. However, we acknowledge that the evaluation of downstream mTOR targets such as p70S6K and 4E-BP1 would provide more comprehensive evidence for mTOR pathway modulation.

Figure 4C demonstrates that, on PC12 cells, compared with the CTRL group, the phosphorylation expression levels on the Thr183 and Thr172 of the α subunit of the AMPK protein in the CLE group were higher. Compared with the MPP^+^ group, the phosphorylation expression levels on Thr183 and Thr172 on the α subunit of the AMPK protein in the MPP^+^+CLE group were higher. These results showed that CLE could increase AMPK phosphorylation and could also increase AMPK phosphorylation in the PC12 treated with MPP^+^. Combined with the cell survival rate analysis in (Figure 6C), compared with the CTRL group, there was no significant change in the cell survival rate of the CLE group. Compared with the MPP^+^ group, the PC12 cells survival rate in the MPP^+^+CLE group was higher. These results indicated that CLE did not affect the cell survival rate of PC12 cells, and to a certain extent, reduced the toxicity of MPP^+^ to PC12 cells, thereby increasing the cell survival rate. The modest improvement in cell viability in the PC12 model may reflect the acute and severe nature of MPP^+^ toxicity, which overwhelms partial autophagy enhancement. In contrast, the chronic MPTP model better reflects the progressive nature of PD and allows for the cumulative effects of CLE treatment. Furthermore, the neuroprotective effect *in vivo* may involve additional mechanisms beyond direct cell survival, including neuroinflammation modulation and synaptic protection, which are not fully captured in the *in vitro* system. The combination of reduced α-syn accumulation, preserved TH^+^ neurons, and behavioral improvement collectively supports the therapeutic potential of CLE. The modest improvement in cell viability *in vitro* should be interpreted cautiously, as it may reflect the partial nature of autophagy enhancement and the multifactorial toxicity of MPP^+^, which affects multiple cellular pathways beyond autophagy.

Consistent with the data in Figure 4B, compared with the MPP^+^ group, the phosphorylation expression level of mTOR Ser2448 in the MPP^+^+CLE group was lower, which could lead to enhanced autophagy downstream. The activation of autophagic flux is the basis for cellular autophagy to exert its biological role. Many studies have shown that neurodegenerative diseases are closely related to the damage of autophagic flow. The intracellular autophagic flux is a process composed of multiple steps and constantly changing. In order to accurately evaluate the situation of autophagic flow, precise experiments need to be conducted. The use of late-stage autophagy inhibitors and the detection of the transformation ratio from LC3-I to LC3-II by WB can reflect the formation level of autophagosomes and is one of the gold indicators for autophagic flow detection. LC3 is a protein widely used to label the membranes of autophagosomes. It exists in the cytoplasm in the form of LC3-I. After stimulation, LC3-II binds to phosphatidylethanolamine and is then located on the inner and outer membranes of autophagosomes. Therefore, the LC3-II/I ratio can be used as an indirect indicator of autophagic flow. When the degradation of autophagosomes in cells is blocked by the late-stage autophagy inhibitor Baf-A1, the change of LC3-II is only an indicator of the change in the number of autophagosomes.

When the autophagic flux of the cell is activated, then under the action of Baf-A1, LC3-II will further increase. If the cell autophagic flow is suppressed, then under the action of Baf-A1, LC3-II will not change. The results presented in Figure 4E demonstrate that the LC3-II in the Baf-A1+CLE group was significantly higher than that in the Baf-A1 group using Baf-A1 alone, indicating that CLE can promote the formation of autophagosomes. To further determine whether CLE affects the degradation of autophagosomes, based on the protein levels of LC3-II/LC3-I, it was found that the difference between the Baf-A1+CLE group and the CLE group was significantly greater than that between the Baf-A1+CLE group and the Baf-A1 group. It indicates that CLE can increase the degradation of autophagosomes. In addition, to initiate the autophagy process, two key steps must be completed: the identification of the substrate to be degraded during the autophagy process and the transportation of the substrate to be degraded into the autophagosome. And autophagic receptors are precisely the key to regulating these steps. p62 is the most important selective autophagic receptor, also known as the selective autophagic receptor, which can link LC3 and ubiquitinated substrates to be degraded. Therefore, the level of p62 protein is negatively correlated with the accumulation of the intracellular polyubiquitinated substances. The expression level of p62 protein detected by WB can reflect the degradation level of autolysosomes.

Figure 4H demonstrates that, compared with the CTRL group, the p62 level of the MPP^+^ group is higher. Compared with the MPP^+^ group, the p62 level in the MPP^+^+CLE group was lower, indicating that CLE may accelerate the degradation of autophagosomes by promoting the fusion between autophagosomes and lysosomes. Autophagy is an intracellular degradation process that forms a double-membrane structure (autophagic vesicle) in the cytoplasm to enclose substances to be cleared (such as damaged organelles or toxic proteins) and transport them to lysosomes for degradation. Lysosomes are single-layer membrane vesicles capable of decomposing various biological macromolecules. They contain multiple hydrolases and can break down substances such as proteins, nucleic acids, polysaccharides, and lipids (Tancini et al., 2020). Autophagosomes refer to newly formed autophagosomes fused autophagosomes (Button and Luo, 2017). autolysosomes refer to the fusion of lysosomes or other endocytic vesicles (such as early endosomes or late endosomes) and autophagosomes (Zhao et al., 2021). To explore the related mechanism of increased autophagy in PC12 cells treated with MPP^+^ by CLE, PC12 cells in the CTRL group, MPP^+^ group, CLE group and MPP^+^+CLE group were stained with LC3 (red), LAMP1 (green) and DAPI (blue) (Figure 5A). Among them, the definition of lysosomal quantification is the spots positive for LAMP1 and negative for LC3, and the definition of autophagosomes is the spots negative for LAMP1 but positive for LC3. The definition of autophagolysosomes is double positive spots for LAMP1 and LC3.

The results presented in Figure 5B showed that, compared with the CTRL group, the number of lysosomes in the MPP^+^ group and the MPP^+^+CLE group decreased. Studies have shown that MPP^+^ has a certain degree of influence on the function and quantity of lysosomes. Exposure to MPP^+^ reduces the activity of lysosomal hydrolase Cathepsin D (Wu et al., 2019), alters the acidity and density of lysosomes, and leads to a decrease in the number of lysosomes (Jung et al., 2019). Consistent with the data in Figure 5B, compared with the CTRL group, the number of lysosomes in CLE did not change significantly. Compared with the MPP^+^ group, the number of lysosomes in the MPP^+^+CLE group increased. The results indicate that CLE partially restored the MPP^+^-induced reduction in lysosome number in PC12 cells. This restoration may improve autophagosome-lysosome fusion and lysosomal degradation capacity, although the additional experiments would be required to directly assess fusion efficiency. CLE may improve the lysosomal function by restoring lysosome numbers, as suggested by the increased autolysosome formation observed in the MPP^+^+CLE group.

Figure 5C demonstrates that, compared with the CTRL group, the number of autophagosomes increased in the MPP^+^ group, the CLE group and the MPP^+^+CLE group. Compared with the MPP^+^ group, the number of autophagosomes in the MPP^+^+CLE group increased. The results presented in Figure 5D showed that, compared with the CTRL group, the number of autophagolysosomes (LC3^+^LAMP1^+^ puncta) increased in the MPP^+^ group, the CLE group and the MPP^+^+CLE group. The results indicated that MPP^+^ activated autophagy to a certain extent, possibly due to the enhanced responsiveness of PC12 cells to autophagy. The impairment of lysosomal function and the disorder of autophagy function are affected by MPP^+^ to a certain extent. CLE partially restored the MPP^+^-induced reduction in lysosome number and was associated with increased autolysosome formation (LC3^+^LAMP1^+^ puncta), suggesting that CLE may improve autophagosome-lysosome fusion. The increased number of autolysosomes (LC3^+^LAMP1^+^ puncta) in CLE-treated cells suggests enhanced autophagosome-lysosome fusion, although we acknowledge that this interpretation is based on indirect measurements. This co-localization could also reflect increased autophagosome formation or altered lysosomal dynamics rather than improved fusion efficiency per se. The direct assessment of fusion would require additional approaches such as live-cell imaging, proximity ligation assays, or electron microscopy to definitively establish whether CLE enhances the fusion process itself.

Pathway inhibitors are compounds that can interfere with or block specific signal transduction pathways. Exploring the functions and mechanisms of a certain signal pathway in cells or organisms provides candidate molecules and targets for the development of new drugs. DOR is an effective AMPK inhibitor. It can not only reveal the regulatory mechanism of the AMPK signaling pathway, but also identify the components and interactions of the signaling pathway, and thereby evaluate the function of this signaling pathway in various diseases and the potential for drug development (Benito-Cuesta et al., 2021). To further determine whether the AMPK/mTOR signaling pathway is the target of CLE and whether CLE can improve the level of α-syn protein in PC12 cells through this signaling pathway, the expression of related proteins such as this signaling pathway was detected by the WB method.

The results presented in Figure 6F demonstrate that, compared with the CTRL group, the phosphorylation expression level at the AMPK Thr183 and Thr172 site in the DOR group and the DOR+CLE group was lower, while the phosphorylation expression levels at AMPK Thr183 and Thr172 sites in the CLE group were higher. Consistent with the data in Figure 6H, compared with the DOR group, the phosphorylation expression levels at AMPK Thr183 and Thr172 sites in the DOR+CLE group is higher. The results presented in Figure 6E show that, compared with the CTRL group, the phosphorylation expression level of mTOR Ser2448 in the DOR group was higher, while that in the CLE group was lower. However, there was no significant difference in the phosphorylation expression level of mTOR Ser2448 in the DOR+CLE group. Compared with the DOR group, the phosphorylation expression level of mTOR Ser2448 in the DOR+CLE group was lower. The results showed that DOR could effectively inhibit the protein expression levels of p-AMPK/t-AMPK and p-mTOR/t-mTOR, indicating that DOR could effectively inhibit the activation of the AMPK/mTOR signaling pathway. DOR can reverse the enhancement of the AMPK/mTOR signaling pathway induced by CLE, indicating that CLE has a regulatory effect on this pathway. Figure 6H demonstrates that, compared with the CTRL group, the protein expression levels of α-syn in the DOR group and the DOR+CLE group were higher, while the protein expression level of α-syn in the CLE group was lower. Compared with the DOR group, the protein expression level of α-syn in the DOR+CLE group was lower. Overall, it indicates that after the inhibition of the AMPK/mTOR signaling pathway activated by CLE, the expression level of the pathological marker α-syn protein increases, which may induce the functional impairment and apoptosis of PC12 cells.

The reduction in α-syn levels following CLE treatment correlates with enhanced autophagy markers, suggesting that autophagy-mediated clearance may contribute to this effect. However, we acknowledge that correlation does not establish causation – reduced α-syn levels could also result from decreased synthesis, increased degradation via other pathways (such as the ubiquitin-proteasome system), or other mechanisms. While the correlation with autophagy enhancement is the compelling, definitive demonstration of autophagy-dependent clearance would require additional experiments using autophagy inhibitors to determine whether CLE’s effect on α-syn is abolished when autophagy is blocked (see Limitations section for further discussion).

To explore whether the results on PC12 were universal or feasible at the animal level, mice were divided into the CTRL group, the MPTP group, and the MPTP+CLE group, and relevant treatments were given respectively. MPTP is metabolized in the body to produce MPP^+^. After MPP^+^ enters the mitochondria, it inhibits the activity of oxidative respiratory chain complex I, causing oxidative stress injury in DaNs. A reduction in the number of DaNs can lead to motor dysfunction. The enhanced phosphorylation of α-syn caused by MPTP is related to the pathogenesis of PD (Li et al., 2020). It can form misfolded and aggregated fibrous structures and spread between cells, leading to neuronal dysfunction and death. There are numerous phosphorylation sites of α-syn, but they play different roles. S129 is the most common and important phosphorylation site, the site is a highly toxic post-translational modification of proteins, which can be phosphorylated by various kinases such as GSK-3β, PLK2, PLK3, etc., increasing the misfolding, polymerization and aggregation of α-syn, leading to the damage of DaNs, and is associated with neurodegenerative diseases such as PD. The Y39 site can be phosphorylated by c-Abl kinase, affecting the lipid binding ability of α-syn and neurotoxicity (Grassi et al., 2018). The Y125 site can be phosphorylated by Src kinase or c-Abl kinase, affecting the distribution and transport of α-syn within cells (El Turk et al., 2018).

The S87 site can be excited phosphorylation by keratin 2 enzyme to promote the polymerization of α-syn and fiber formation (Levine et al., 2019). To explore whether CLE could improve the p-α-SYN protein level of mouse DaNs, it was verified in immunofluorescence staining (Figure 8C). The results presented in Figure 8D show that, compared with the CTRL group, the p-α-SYN protein levels in the MPTP group and the MPTP+CLE group were higher. Compared with the MPTP group, the protein expression level of p-α-SYN in the MPTP+CLE group was lower. The results indicated that CLE could effectively improve the increased p-α-SYN protein level after the MPTP treatment of DaNs. In addition, MPTP increases the α-SYN protein in the midbrain of mice and the p-α-SYN protein in DaNs, sensitively activates the AMPK/mTOR pathway, and raises the autophagy level, but it is not sufficient to completely clear the α-SYN protein and the p-α-SYN protein in DaNs.

CLE can compensate for the insufficient activation of the AMPK/mTOR pathway, increase the level of autophagy, and improve the clearance ability of α -SYN protein in the midbrain of mice and p-α-SYN protein in DaNs. The motor symptoms caused by MPTP refer to the fact that MPTP can damage the DaNs of mice, resulting in a decline in the motor balance ability of mice. In the rotating rod experiment, it is manifested as a shortened stay time on the constantly rotating rod and an increased number of drops (Zhang et al., 2019). The rod-spinning test is one of the classic methods for detecting motor dysfunction in mice and is also a common indicator for evaluating the symptoms of PD in MPTP model mice. When DaNs are damaged or reduced, it leads to a decrease in the level of dopamine in the brain, thereby affecting the motor regulatory circuits of the basal ganglia. CLE can effectively alleviate the motor balance ability disorder in mice caused by MPTP, which may be due to the protective effect of CLE on DaNs.

There is evidence suggesting that constipation occurs early in PD regarding motor dysfunction. Constipation, as a common non-motor symptom, troubles many PD patients. Its occurrence mechanism involves multiple aspects, including intestinal neurodegeneration, drug effects, dietary habits and anorectal dysfunction, etc. The intestinal nervous system of PD patients is affected by the pathological deposition of α-syn, resulting in slowed intestinal peristalsis and delayed colonic transport. The dietary habits and insufficient water intake of PD patients may lead to dry and hard stools, increasing the difficulty of defecation (De Pablo-Fernández et al., 2019). The anorectal dysfunction in PD patients, such as decreased the anorectal sensation, the abnormal defecation reflex, and reduced sphincter tension, may lead to obstruction or incomplete defecation (De Pablo-Fernández et al., 2019). The experimental results showed that MPTP could also cause constipation-like symptoms. Studies have shown that chronic low-dose MPTP increases the expression level of α-syn protein in the ileum of mice, causes intestinal inflammation in mice, damages the intestinal barrier of mice, affects the composition of the intestinal microbiota of mice, and leads to constipation-like symptoms (Lai et al., 2018). Therefore, MPTP can cause something similar to the non-motor symptoms of constipation in PD patients with chronic diseases. The treatment of CLE alleviated the non-motor symptoms of constipation-like in mice caused by MPTP, possibly because CLE has the potential to alleviate the overexpression of α-syn protein in the ileum of mice induced by MPTP. CLE also ameliorated constipation-like symptoms, increasing both fecal pellet count (Figure 9D) and fecal water content (Figure 9E). While these findings suggest improvement in non-motor symptoms, we acknowledge that the definitive demonstration of PD-associated constipation-like symptoms improvement would require additional gastrointestinal mechanistic evidence, such as the assessment of intestinal α-syn pathology, the enteric neuronal survival, or the colonic transit time.

To sum up, this study revealed the multi-level protective effects of CLE on the PD model, including the activation of the AMPK/mTOR signaling pathway, the enhancement of autophagic flux, the reduction of pathological proteins α-syn and p-α-syn (likely through the autophagy-mediated clearance), the protection of PC12 cells and DaNs, and the alleviation of motor and non-motor symptoms. While these findings are the suggestive of a causal relationship between autophagy enhancement and α-syn clearance, further studies with autophagy inhibitors are needed to definitively establish this mechanism. These findings provide new mechanisms and evidences for CLE as a potential drug for treating PD. However, this research was limited to verification at the cellular and animal levels and can’t fully reflect the complex and changeable situations at the human level. In subsequent research, in order to deal with the complex and changeable situations at the human body level, the research on the direct interaction between CLE and target proteins will be improved based on the results of molecular docking and related bioinformatic analyses.

## Limitations

We acknowledge the important limitations of our experimental models. PC12 cells, while widely used in PD research, are derived from rat pheochromocytoma and do not fully reproduce the complex biology of human nigral DaNs. Additionally, the MPP^+^-induced PC12 cell model and chronic MPTP mouse model represent toxin-based models that do not fully recapitulate the idiopathic nature, progressive neurodegeneration, or the full spectrum of pathological features observed in human PD, including the age-dependent onset and the involvement of multiple cell types. Therefore, our findings, while providing valuable mechanistic insights, should be interpreted with appropriate caution when extrapolating to human PD.

The single-cell RNA-seq analysis has limitations, including the small number of human donors (5 PD, 6 HCs) and the limited number of DaNs identified (74 cells). Additionally, the DEG analysis treated individual cells as the statistical unit, which may introduce pseudoreplication. Future studies employing donor-level/pseudobulk approaches with larger sample sizes would strengthen the statistical robustness of these findings.

The molecular docking analysis, while providing valuable hypothesis-generating data, does not establish direct drug-target interactions. Docking scores and binding energies are predictive and require experimental validation through techniques such as surface plasmon resonance, the isothermal titration calorimetry, or the cellular thermal shift assays to confirm binding. Therefore, the identified targets should be considered putative rather than confirmed.

Molecular docking was used as a hypothesis-generating tool to predict the potential protein targets of CLE. The docking results should be interpreted as suggestive rather than definitive, as they require experimental validation to establish direct drug-target interactions.

We acknowledge that our assessment of mTOR activity is limited to the phosphorylation of mTOR at Ser2448. While this site is a well-established marker of mTORC1 activity, the evaluation of downstream targets such as p70S6K and 4E-BP1 would provide more comprehensive evidence for mTOR pathway modulation. Therefore, our conclusions regarding reduced mTOR activity should be interpreted with this limitation in mind. While the evaluation of mTOR Ser2448 phosphorylation provides evidence for reduced mTOR activity, the examination of downstream targets such as p70S6K and 4E-BP1 would provide more comprehensive validation and should be addressed in future studies.

We acknowledge that while dorsomorphin provides pharmacological evidence for AMPK involvement, genetic validation using AMPK or mTOR knockout/knockdown approaches would be required to definitively establish pathway specificity. Therefore, our conclusions regarding pathway-specific mechanisms should be interpreted with appropriate caution.

While our data suggest a correlation between autophagy enhancement and α-syn reduction, we acknowledge that the definitive demonstration of autophagy-dependent α-syn clearance would require additional experiments, such as co-treatment with autophagy inhibitors (e.g., chloroquine, 3-MA, or Baf-A1) to determine whether CLE’s effect on α-syn is abolished when autophagy is blocked. Therefore, our interpretation of autophagy-dependent α-syn clearance should be considered suggestive rather than conclusive.

Additionally, while our data suggest a correlation between autophagy enhancement and α-syn reduction, we acknowledge that the definitive demonstration of autophagy-dependent α-syn clearance would require additional experiments, such as co-treatment with autophagy inhibitors to determine whether CLE’s effect on α-syn is abolished. Furthermore, our assessment of autophagosome-lysosome fusion is limited to the indirect measurements of autolysosome formation (LC3^+^LAMP1^+^ co-localization). Additional approaches would be needed for definitive conclusions about fusion efficiency.

While these findings are the suggestive of enhanced autophagic flux, we acknowledge that the definitive demonstration of complete autophagic flux would require additional approaches such as tandem fluorescent-tagged LC3 (GFP-mRFP-LC3) assays or electron microscopy to directly visualize autophagosome-lysosome fusion and degradation.

## Data Availability

The original contributions presented in the study are included in the article/Supplementary material, further inquiries can be directed to the corresponding author.
