# Supplementary material for: Clemastine alleviates Parkinson’s disease pathology by activating the AMPK/mTOR pathway and enhancing autophagic flux
Source: Front Aging Neurosci. 2026 Sep 29;18:1915475. doi: 10.3389/fnagi.2026.1915475 (PMC13658160; doi:10.3389/fnagi.2026.1915475)
Supplement: Supplementary file 1 [file Data_Sheet_1.docx]

### **Supplementary Table 1. Categorization of Autophagy-Related Targets of Clemastine by Functional Role**

This table categorizes the autophagy-related protein targets of clemastine (identified through molecular docking) and key autophagy regulators discussed in the manuscript, based on their primary functional roles in the autophagy-lysosome pathway.

| **Functional Category** | **Target Protein** | **Role in Autophagy & Pathway** |
| --- | --- | --- |
| **1. Core Autophagy Regulators & Signaling** | **AMPK** | A key energy sensor and positive regulator of autophagy. It activates autophagy by inhibiting the mTORC1 pathway and directly activating the ULK1 complex. |
|  | **mTOR** | A central negative regulator of autophagy. It inhibits the initiation of autophagy by phosphorylating and inactivating the ULK1 complex. |
|  | **ATG4C** | A cysteine protease involved in the processing of LC3 (cleaving pro-LC3 to LC3-I) and the de-conjugation of LC3-II from the autophagosome membrane, which is essential for autophagosome formation and recycling. |
|  | **ULK1** | (Implied by pathway) A serine/threonine kinase that is a key initiator of autophagy. It is activated by AMPK and inhibited by mTORC1. |
|  | **VPS34** | (Implied by pathway) A class III PI3 kinase that is crucial for the nucleation and formation of the autophagosome membrane. |
| **2. Autophagosome Formation & Elongation** | **LC3 (MAP1LC3)** | A ubiquitin-like protein that is conjugated to phosphatidylethanolamine (forming LC3-II) and incorporated into the autophagosome membrane. It is a widely used marker for autophagosomes and is essential for autophagosome formation and cargo selection. |
|  | **Beclin-1** | (Implied by pathway) A core component of the VPS34-PI3K complex that is essential for the initiation of autophagosome formation. |
| **3. Substrate Recognition & Selective Autophagy** | **p62 (SQSTM1)** | A selective autophagy receptor (autophagic cargo protein) that links ubiquitinated substrates (like α-synuclein) to the autophagosome machinery by binding to both ubiquitin and LC3. Its degradation is a marker of autophagic flux. |
|  | **NFE2L2 (NRF2)** | A transcription factor that can be regulated by the autophagy substrate p62. It is a master regulator of the antioxidant response, and its dysfunction is linked to impaired autophagy and proteostasis. Its strong correlation with Braak staging suggests a link to autophagy dysfunction and PD severity. |
| **4. Lysosomal Function & Fusion** | **LAMP1** | A major lysosomal membrane protein. It serves as a marker for lysosomes and is crucial for lysosomal integrity and fusion with autophagosomes to form autolysosomes. |
|  | **LAMP-2A** | (Discussed in Introduction) A lysosomal receptor protein that is essential for chaperone-mediated autophagy (CMA). |
|  | **Cathepsin D** | (Discussed in Discussion) A major lysosomal hydrolase. Its activity is critical for the degradation of proteins (including α-synuclein) within the autolysosome. MPP+ was shown to reduce its activity. |
|  | **NFKB1** | A subunit of the NF-κB transcription factor. It is involved in the inflammatory response, and its signaling pathway can interact with and modulate autophagy. Its correlation with Braak staging indicates a possible connection between inflammation, autophagy, and disease progression. |
| **5. Vesicle Trafficking & Autophagosome Maturation** | **MTMR14** | A myotubularin-related protein that functions as a phosphoinositide 3-phosphatase. It is involved in the regulation of membrane trafficking and the autophagic process, potentially affecting autophagosome maturation or fusion. |
|  | **RAB7** | (Implied by pathway) A small GTPase that is essential for the fusion of autophagosomes with lysosomes. |
| **6. α-Synuclein Degradation** | **α-Synuclein (α-syn)** | The primary pathological protein in PD. It is a substrate for autophagy, and its clearance by the autophagy-lysosome pathway (both macroautophagy and CMA) is a central focus of the study. |

**Note:** Proteins marked as "(Discussed in Introduction/Discussion)" or "(Implied by pathway)" are not direct docking targets listed in Tables 3 and 4 but are key players in the autophagy pathway discussed in the manuscript's text. Targets listed in the table are primarily from the molecular docking results (Tables 3 and 4) unless otherwise indicated.
